# Deciphering the fine nucleotide diversity of full HLA class I and class II genes in a well‐documented population from sub‐Saharan Africa

**DOI:** 10.1111/tan.13180

**Published:** 2017-12-25

**Authors:** T. Goeury, L. E. Creary, L. Brunet, M. Galan, M. Pasquier, B. Kervaire, A. Langaney, J.‐M. Tiercy, M. A. Fernández‐Viña, J. M. Nunes, A. Sanchez‐Mazas

**Affiliations:** ^1^ Laboratory of Anthropology, Genetics and Peopling History, Department of Genetics and Evolution ‐ Anthropology Unit University of Geneva Geneva Switzerland; ^2^ Institute of Genetics and Genomics in Geneva University of Geneva Geneva Switzerland; ^3^ Department of Pathology Stanford University School of Medicine Palo Alto California; ^4^ Transplantation Immunology Unit and National Reference Laboratory for Histocompatibility (UIT/LNRH) Geneva University Hospital Geneva Switzerland; ^5^ INRA, UMR 1062 CBGP avenue du Campus Agropolis Montferrier sur Lez France

**Keywords:** allelic conversion, balancing selection, full‐length HLA genes, HLA nucleotide diversity, *Onchocerciasis*, population genetics, selective sweep, West Africa

## Abstract

With the aim to understand how next‐generation sequencing (NGS) improves both our assessment of genetic variation within populations and our knowledge on HLA molecular evolution, we sequenced and analysed 8 HLA loci in a well‐documented population from sub‐Saharan Africa (Mandenka). The results of full‐gene NGS‐MiSeq sequencing compared with those obtained by traditional typing techniques or limited sequencing strategies showed that segregating sites located outside exon 2 are crucial to describe not only class I but also class II population diversity. A comprehensive analysis of exons 2, 3, 4 and 5 nucleotide diversity at the 8 HLA loci revealed remarkable differences among these gene regions, notably a greater variation concentrated in the antigen recognition sites of class I exons 3 and some class II exons 2, likely associated with their peptide‐presentation function, a lower diversity of HLA‐C exon 3, possibly related to its role as a KIR ligand, and a peculiar molecular diversity of HLA‐A exon 2, revealing demographic signals. Based on full‐length HLA sequences, we also propose that the most frequent DRB1 allele in the studied population, DRB1*13:04, emerged from an allelic conversion involving 3 potential alleles as donors and DRB1*11:02:01 as recipient. Finally, our analysis revealed a high occurrence of the DRB1*13:04‐DQA1*05:05:01‐DQB1*03:19 haplotype, possibly resulting from a selective sweep due to protection to *Onchorcerca volvulus*, a prevalent pathogen in West Africa. This study unveils highly relevant information on the molecular evolution of HLA genes in relation to their immune function, calling for similar analyses in other populations living in contrasting environments.

## 1 INTRODUCTION

Due to the extreme polymorphism of the HLA genomic region^1^ (www.ebi.ac.uk/ipd/imgt/hla/stats.html), the application of next‐generation sequencing (NGS) to HLA genes has been particularly challenging in the last decades, requiring careful tests and comparisons among different competing technologies.^2^, ^3^, ^4^, ^5^, ^6^, ^7^, ^8^ However, thanks to tremendous efforts motivated by the need of tissue‐typing laboratories to improve the accuracy and throughput of HLA genotyping of potential donors and patients, new HLA sequencing platforms are currently being implemented in most countries, leading to both a rapid discovery of new alleles (eg, 17% increase of the total number of alleles and 119% of the total number of alleles with complete sequences between April 2016 and April 2017, www.ebi.ac.uk/ipd/imgt/hla/stats.html, see also, for example, Reference 9) and a better characterisation of multi‐locus haplotypes which are expected to improve the HLA data quality of hundreds of donor registries throughout the world.

Besides its potential benefits for histocompatibility, DNA sequencing of the HLA region also opens new perspectives in the area of human population genetics by permitting direct analyses of nucleotide variation within a molecular evolutionary genetics framework.^10^, ^11^, ^12^ Although stimulating results on human populations' molecular diversity were obtained previously by inferring sequence genotypes to large sets of population samples thanks to the molecular information stored in the IMGT‐HLA database,^13^ such approaches could only use data defined at the second field level of resolution, thus ignoring the information provided by synonymous substitutions and by regions located outside exons 2 and 3. As NGS established itself very recently as the next HLA typing standard,^14^ we thus decided to use NGS techniques to decipher the fine nucleotide diversity of 8 HLA genes in a well‐documented human population.

Our primary goal in this study was to understand better the relationships between HLA nucleotide variation and different demographic and selective forces which could have driven the molecular evolution of the HLA loci. We thus chose to analyse a population that was already well‐known from both an anthropological (*lato sensu*) and a genetic point of view. The Mandenkalu (plural of Mandenka) were sampled during a field study undertaken in the 1990s in Eastern Senegal, West Africa, after several previous expeditions which documented the demography and peopling history of this region (see Reference 15 for a review). Several genetic polymorphisms were then analysed, among which immunoglobulin markers,^16^, ^17^, ^18^ HLA by both serological^16^ and Polymerase Chain Reaction‐Sequence Specific Oligonucleotide (PCRSSO)^19^ methods, mtDNA,^20^ genome‐wide Restriction Fragment Length Polymorphisms (RFLPs),^21^ alpha‐22 and beta‐globins^23^ and N‐acetyltransferase 2.^24^ Based on these different sources of independent information, the Mandenka population (which is also since many years a reference population in the HGDP‐CEPH Database, www.cephb.fr/hgdp/main.php), is known to exhibit a very high level of genetic diversity, probably as a result of population expansion,^25^ and is considered to be representative of a larger population group of Western Africa.^23^

Interestingly, these results allow the exclusion of genetic drift as a major evolutionary factor shaping the molecular profile of this population, thus providing an ideal framework to explore the effects of natural selection on the HLA genes. Such effects, which result from the crucial immunological function played by the HLA molecules, are indeed generally difficult to disentangle from those of demography.^26^ Although linear modelling or comparisons with simulated data may be successfully used in this perspective^27^, ^28^, ^29^, such approaches need large sets of HLA‐typed population samples, which are currently only available in the form of first‐field HLA allelic frequencies. Moreover, different types of selective pressures have been invoked to affect the HLA region (see Reference 30 for a review). Although balancing selection in the form of heterozygous advantage (more particularly under the *divergent allele advantage* model^31^) is generally considered as the main mechanism explaining the very high level of polymorphism observed at these genes in most human populations,^32^ positive selection increasing the frequency of alleles putatively protective to specific pathogens^29^, ^33^, ^34^, ^35^, ^36^ as well as *joint divergent asymmetric selection* involving simultaneous contributions of several HLA loci^37^ have also been proposed to explain the lower diversity observed in some populations at specific HLA loci. In this context, the analysis of the Mandenka population is particularly motivating because it lives in a region where several infectious diseases are highly prevalent. In addition, although sub‐Saharan African populations, which represent more than 2000 ethno‐linguistic groups,^38^ have been extensively studied for different genetic markers^39^, ^40^, ^41^, ^42^, ^43^, ^44^ including HLA,^29^, ^45^, ^46^, ^47^, ^48^ to our knowledge their HLA molecular variability has not been analysed so far at its greatest degree of detail, that is, the nucleotide level. In this study, we thus investigated the nucleotide polymorphism of 8 extended HLA genes in a sample of the Mandenka population by using full‐gene NGS‐MiSeq high‐throughput sequencing. To evaluate the advantages of using this technology, we first compared the NGS‐MiSeq results with those of a traditional typing technique (PCR‐SSO on exons 2/3) and a limited sequencing strategy (NGS‐454 on exon 2) applied to the same population sample. For the purpose of comparing the typing technologies we used all individuals that were typed with those techniques, hence using the maximum amount of data available, and for the subsequent population genetics analyses we used only subsets of unrelated individuals. In this context, we estimated several molecular population diversity indexes and compared them among different gene regions within and between 8 HLA loci by using both the nucleotide and inferred amino acid information. We finally discussed our results in relation to several hypotheses of natural selection affecting different HLA gene regions and loci.

## 2 MATERIALS AND METHODS

### 2.1 Population sample

The Western African Mandenka population speaks a language belonging to the Mande branch of the Niger‐Congo family (widely represented among African‐Americans nowadays^49^). During a sampling campaign performed in January to February 1990, 20 mL of peripheral blood was taken from 205 informed volunteers living in 5 Mandenka villages in the Bandafassi district near Kédougou (Supplementary Information S01). Pedigree relationships between individuals were also recorded, and 101 of them could be considered as unrelated.

### 2.2 HLA typings

In a time span of about 25 years, the same Mandenka population sample was HLA typed by using 3 different techniques: a traditional typing technology (PCR‐SSO on exons 2/3), whose results were reported between 1992 and 2007,^19^, ^50^, ^51^ a limited sequencing strategy (NGS‐454 on exon 2, only applied to class II loci) and full‐gene NGS‐MiSeq sequencing, the results of both NGS‐454 and NGS‐MiSeq being reported here for the first time. Of the initial set of 205 sampled individuals, 165 to 198 individuals (depending on the locus) were successfully typed by PCR‐SSO for the 6 loci A, B, C, DRB1, DQB1 and DPB1, 194 to 199 individuals by NGS‐454 for the 4 class II loci DRB1, DQA1, DQB1 and DPB1 and 51 to 86 individuals by NGS‐MiSeq for the 8 loci A, B, C, DRB1, DQA1, DQB1, DPB1 and DPB1. These HLA‐typings, for which complete descriptions can be found in Supplementary Information S01, are summarized below.

### 2.3 PCR‐SSO typings of class I (exons 2 and 3) and class II (exon 2) genes

HLA‐DRB1, DQB1 and DPB1 typings were performed by locus‐ and group‐specific PCR amplification, followed by direct hybridization with sequence‐specific oligonucleotide (SSO) probes on nylon filters. For HLA class I DNA typings, samples were first tested by direct PCR‐SSO hybridization using locally designed probes^52^, ^53^, ^54^ and were further retyped by using the reverse PCR‐SSO hybridization protocol of the 13th International Histocompatibility and Immunogenetics Workshop.^51^

### 2.4 NGS‐454 sequencing of class II genes (exon 2)

Tagging and multiplexing methods designed by Galan et al^55^ for Roche NGS‐454 sequencing were used. For this high‐throughput sequencing, adaptors are required for the emPCR and 454 GS‐FLX pyrosequencing using Lib‐L Titanium Series reagents. Four HLA Class II genes were amplified and sequenced using this method (DRB1, DQA1, DQB1, DPB1). For each gene, 245 samples (+12 control H2O) were amplified. A total of 46 replicates (19%) were used to confirm the protocol. Sequencing was performed by Beckman Coulter Genomics (Genomic Services, Danvers, Massachuetts). Reads were filtered using Mothur^56^ with a minimal PhredScore of 30. These data were explored using SESAME Barcode.^57^

### 2.5 NGS‐MiSeq sequencing of full class I and class II genes

Two complementary techniques were used in 2 different laboratories to sequence full HLA class I and class II genes:

- *Geneva University Hospital*: Fifty‐four individuals were typed by using the Holotype HLA X2 kit (Omixon Biocomputing Ltd, Budapest, Hungary) in combination with the Illumina MiSeq platform to type the 7 HLA loci A, B, C, DRB1, DQA1, DQB1 and DPB1. Sequences generated by MiSeq were processed by the software HLA Twin v1.1.1 (Omixon, Biocomputing Ltd, Budapest, Hungary).
- *Stanford University*: Sixty‐five individuals (25 among the 54 individuals sequenced at Geneva University Hospital to confirm uncertain sequences, plus 40 additional individuals) were typed for the 8 HLA loci A, B, C, DPA1, DPB1, DQA1, DQB1, DRB1 using the MIA FORA NGS typing method developed by Sirona Genomics (Immucor, Inc, Norcross, Georgia) and performed following the manufacturer's instructions. Alleles were assigned using the Sirona Genomics NGS alignment software which uses 2 complementary informatic strategies to make genotyping calls. The first strategy utilizes Expectation Maximization to rank computed allele candidates based on mapping metrics. Coverage is calculated from competitive alignment of paired‐end NGS reads with all HLA reference sequences in the IMGT/HLA database 3.22.0^58^ and internal references generated by cloning and sequencing. The second strategy utilizes a dynamic phasing algorithm to assemble reads and construct one or 2 phased consensus sequences by de novo assembly of mapped paired‐end sequences. These consensus sequences are then aligned to the HLA allele database to find the best fit.

Unambiguous genotypes composed of 2 phased sequences were finally obtained for 51 to 86 different individuals (as mentioned above in HLA typings), depending on the locus (the precise numbers per locus are given in Tables 2 and 3). Only a few alleles (1.28%) were found to be ambiguous. In those cases, we kept the list of all possible alleles to perform the analyses.

### 2.6 NGS‐MiSeq sequence alignments

Consensus sequences for each individual and each locus were retrieved from NGS‐MiSeq sequencing. Sequences were aligned against a reference sequence (from IMGT/HLA^58^ v3.25.00) using MAFFT^59^ and exons were mapped onto the alignments using MAFFT “*‐‐add*” option. Table 1 lists the different reference sequences used. Alignments were checked individually to remove divergent and badly aligned sequences. From these alignments, gene regions (corresponding to the “gene features” defined by Mack 2015,^60^ and ranging from 5'UTR to 3'UTR when available) were extracted separately for each sequenced individual.

**Table 1:** Reference sequences used for the alignments of the 8 HLA loci and their gene regions

Locus	Reference gene	Reference exon	Number of exons	N Seqs
A	A*01:01:01:01	A*01:01:01:01	8	174
B	B*07:02:01	B*07:02:01	7	166
C	C*01:02:01	C*01:02:01	8	166
DRB1	DRB1*01:01:01	DRB1*01:01:01	6	160
DQA1	DQA1*01:01:02	DQA1*01:01:01:01	4	158
DQB1	DQB1*02:01:01	DQB1*05:03:01:01	6	154
DPA1	DPA1*01:03:01:01	DPA1*01:03:01:01	4	102
DPB1	DPB1*02:01:02	DPB1*01:01:01	5	166

N Seqs, number of consensus sequences retrieved after the filtering steps.

### 2.7 Population genetics analyses

#### 2.7.1 Comparison of molecular typing strategies

For each locus, the HLA genotype distributions obtained for the Mandenka individuals by using different molecular typing strategies (PCR‐SSO, NGS‐454 and NGS‐MiSeq) were compared 2 by 2. To that aim, we considered the subsamples of individuals typed by each pair of techniques. In order to be conservative, a good match between 2 techniques was only reported when the 2 alleles of the compared genotype were identical (ie, if at least one allele was different, the comparison was reported as a mismatch).

We also used random sampling to estimate a possible variability in the number of genotype matches due to unequal numbers of individuals used to compare different pairs of techniques (either at the same locus or at different loci). As the smallest number of individuals compared between 2 techniques was 66 (both at HLA‐C when comparing PCR‐SSO and NGS‐MiSeq and at HLA‐DQA1 when comparing NGS‐454 and NGS‐MiSeq), we generated 1000 pairs of random samples of 66 individuals taken without replacement among those who were genotyped by 2 different techniques at each locus and we calculated their match. For each pair of techniques compared at each locus, we then plotted in the same graph the observed number of genotype matches and the distribution of genotype matches generated by this resampling procedure.

#### 2.7.2 Allele and haplotype frequencies and linkage disequilibrium

We then used the subsamples of unrelated individuals typed at different loci and by different typing strategies to estimate allele and haplotype frequencies as well as other basic statistics (ie, number of alleles observed (k), allelic richness (*ar*), heterozygosity (*H*) and number of most frequent alleles reaching a cumulated frequency of at least 50% (*F50*) at each studied locus). HLA allele and haplotype frequencies were estimated by using an Expectation‐Maximisation (EM) algorithm. Global linkage disequilibrium between each pair of loci was tested by both parametric and non‐parametric approaches and individual linkage disequilibrium between the alleles of each haplotype by means of standardised residuals. All analyses were performed with the gene[rate] computer tools.^61^, ^62^ Allelic richness was computed with the rarefaction method,^63^ estimating the number of alleles that would be detected if all sample sizes were as small as the smallest sample size used in the study.

#### 2.7.3 Neutrality tests

For each exon taken separately, Tajima's *D*,^64^, ^65^ the ratio of non‐synonymous to synonymous substitutions d*N*/d*S* 66 and the nucleotide diversity π indexes were computed (Tajima's D and π with Arlequin v3.5 software^67^ and d*N*/d*S* with the MEGA software,^68^ using the Nei‐Gojobori method^69^) both on the whole exon sequence (obtained with NGS‐MiSeq) and on the first, second and third nucleotides of each codon to explore the nucleotide variation across all non‐degenerate and degenerate nucleotides. Codons encoding the antigen recognition site (ARS)^70^ were analysed separately from the other codons (non‐ARS) of exons 2 (for class I and II) and 3 (for class I). Note that, as it was impossible to distinguish putative indels from non‐sequenced positions, all gene regions' sequences with gaps were discarded from the analysis (the only exception being for HLA‐DQA1 exon 2, which includes a well‐known indel tri‐nucleotide polymorphism at codon 56), thus focusing the study only on nucleotide substitutions' variability. A missing level of 5% per site was allowed in Arlequin's computations, whereas MEGA considered the codons with gaps as missing information.

As we performed Tajima's test for selective neutrality, which is 2‐sided, we used “p‐Adj = 2*(1 – p‐Value)” for *P*‐Values above .5 and “*p‐Adj =* 2*p‐Value” for *P*‐Values below .5. The Benjamini‐Hochberg^71^ correction for multiple testing was then applied, as implemented in r. *dN/dS* significance was assessed with the *Z* test, where “Z = (*dN‐dS)/(Var(dN) + Var(dS))*” follows a standard normal distribution under the null hypothesis *H*_*0*_.

As several correlated statistics were computed for several gene regions at several HLA loci (Tajima's D and number of segregating sites S, d*N* and d*S*), we compared the molecular diversity of all sequenced exons 2, 3, 4 and 5 (the latter for class I genes), which were well covered at all studied loci, globally by performing a Principal Component Analysis (PCA) on these statistics.

## 3 RESULTS

### 3.1 Genotype matches between the 3 HLA typing strategies

**Figure 1:**
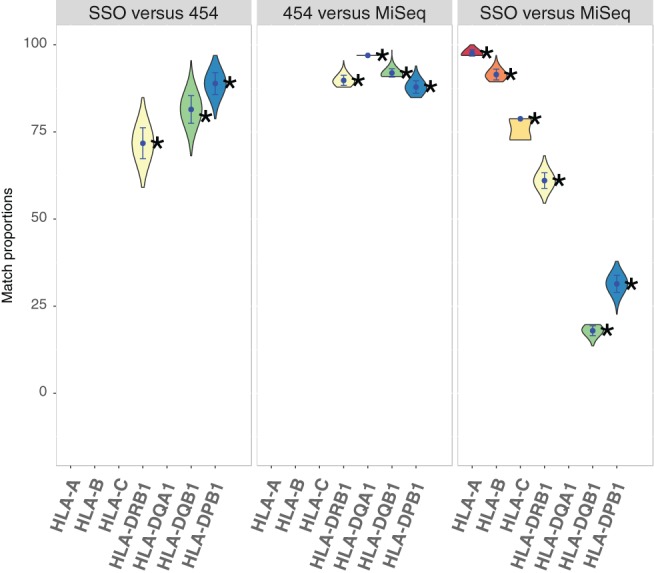
Proportions of genotype matches (or « matching scores ») obtained between PCR‐SSO (“SSO” in the Figure), NGS‐454 (“454”) and NGS‐MiSeq (“MiSeq”) at 3 to 6 HLA loci (DQA1 was not typed with PCR‐SSO and class I genes were not typed with NGS‐454). For the comparisons involving NGS‐454, as the sequences obtained with this technique were limited to exon 2 and may thus correspond to different alleles, we reported a match when the allele found with the other technique was *compatible* with the NGS‐454 sequence. Only perfect matches (i.e. for any compared genotype, when both alleles found with the 2 techniques were either compatible or identical) were counted. The comparisons were replicated 1′000 times each on random samples of 66 individuals (corresponding to the lowest sample size of the observed data) drawn without replacement to assess the variability of genotype matches due to sampling size

Table 2 gives the proportions of genotype matches observed between each pair of typing strategies used to analyse the HLA polymorphism in the Mandenka population. These values are also shown in Figure 1, together with violin plots representing the distributions of the proportion of genotype matches estimated on 1000 random samples of identical size to account for a possible variability due to differences in the number of individuals compared for each pair of techniques (see section 2). The shape and size of these violin plots as well as their position relatively to the observed values indicate that differences in sample sizes occur as expected from a well‐behaved normal sampling distribution, hence ensuring the absence of sampling bias in our results. As a further evaluation of the relative importance of the typing mismatches on the characterisation of the HLA genetic profile of the Mandenka population, we also estimated HLA allele frequencies and basic statistics on the subsamples of unrelated individuals typed at each locus by each technique (Table 3 and Supplementary Information S02).

**Table 2:** Number of genotype matches between PCR‐SSO, NGS‐454 and NGS‐MiSeq

Locus		Pairwise comparisons between different typing techniques					
		PCR‐SSO vs NGS‐454		NGS‐454 vs NGS‐MiSeq		PCR‐SSO vs NGS‐MiSeq	
HLA‐A	No. of genotyped individuals					*196*	*87*
	No. of compared individuals					*85*	
	No. of genotype matches (%)					*83 (97.6%)*	
	CI95					97.0–100.0%	
HLA‐B	No. of genotyped individuals					*198*	*83*
	No. of compared individuals					*82*	
	No. of genotype matches (%)					*75 (91.5%)*	
	CI95					89.4–95.5%	
HLA‐C	No. of genotyped individuals					*165*	*83*
	No. of compared individuals					*66*	
	No. of genotype matches (%)					*52 (78.8%)*	
	CI95					72.7–72.7%	
HLA‐DRB1	No. of genotyped individuals	*198*	*194*	*194*	*81*	*198*	*81*
	No. of compared individuals	*188*		*78*		*77*	
	No. of genotype matches (%)	*135 (71.8%)*		*70 (89.7%)*		*47 (61.0%)*	
	CI95	63.6–80.3%		87.9–92.4%		57.5–65.2%	
HLA‐DQA1	No. of genotyped individuals			*194*	*82*		
	No. of compared individuals			*66*			
	No. of genotype matches (%)			*64 (97.0%)*			
	CI95			97.0%			
HLA‐DQB1	No. of genotyped individuals	*195*	*196*	*196*	*76*	*195*	*76*
	No. of compared individuals	*188*		*74*		*72*	
	No. of genotype matches (%)	*153 (81.4%)*		*68 (91.9%)*		*13 (18.1%)*	
	CI95	72.7–87.9%		90.9–93.9%		15.2–19.7%	
HLA‐DPB1	No. of genotyped individuals	*193*	*199*	*199*	*82*	*193*	*82*
	No. of compared individuals	*193*		*82*		*79*	
	No. of genotype matches (%)	*172 (89.1%)*		*72 (87.8%)*		*24 (30.3%)*	
	CI95	83.3–94.0%		84.9–90.9%		25.8–36.4%	

No., Number; CI95, 95% confidence interval of the percentage of matches estimated by considering that the number of individuals compared between 2 different techniques is as small as the smallest number of individuals actually compared (ie, 66 individuals which is the number compared both for HLA‐C between PCR‐SSO and NGS‐MiSeq and for HLA‐DQA1 between NGS‐454 and NGS‐MiSeq); these intervals were obtained by drawing 1000 random samples of 66 individuals without replacement, see section 2.

**Table 3:** Statistics describing the genetic diversity of the Mandenka population based on HLA molecular typings obtained by 3 different techniques, PCR‐SSO, NGS‐454 and NGS‐MiSeq

Locus	*N* (total)			*N* (unrelated)			*k/ar*			*H* (%)			*F50*		
	PCR‐SSO (second‐field)	NGS‐454	NGS‐MiSeq	PCR‐SSO (second‐field)	NGS‐454	NGS‐MiSeq	PCR‐SSO (second‐field)	NGS‐454	NGS‐MiSeq	PCR‐SSO (second‐field)	NGS‐454	NGS‐MiSeq	PCR‐SSO (seond‐field)	454	NGS‐MiSeq
A	196	–	87	72	–	72	23/21.6	–	22/20.7	92.2	–	92.0	5	–	5
B	198	–	83	67	–	67	30/27.4	–	30/27.8	93.6	–	93.6	6	–	7
C	165	–	83	54	–	54	15/15	–	18/17.9	89.4	–	91.0	4	–	5
DRB1	198	194	81	96	96	65	22/19.0	23/20.3	20/16.9	87.7	87.6	87.6	3	4	4
DQA1	–	194	82	–	66	66	–	9/9	14/13.0	–	60.7	71.5	–	1	1
DQB1	195	196	76	94	96	60	12/10.9	11/10.5	13/12.8	66.2	68.2	76.7	1	1	2
DPA1	–	–	51	–	–	51	–	–	10/10	–	–	71.9	–	–	2
DPB1	193	199	82	99	101	70	18/14.4	14/12.7	19/16.6	80.0	80.7	86.3	2	2	3

*N* (total), total number of individuals analysed for which the typing yielded usable results; *N* (unrelated), number of unrelated individuals analysed for which the typing yielded usable results; *k,* number of alleles detected; ar, allelic richness or number of alleles expected in a population whose size is equal to the smallest *N* used with a given technique (ie, 54 for PCR‐SSO, 66 for NGS‐454 and 54 for NGS‐MiSeq); *H,* heterozygosity; *F50,* number of most frequent alleles whose cumulated frequency reaches at least 50%.

Class I loci were not typed with NGS‐454, DQA1 was not typed with PCR‐SSO, and DPA1 was only typed with NGS‐MiSeq.

Relatively good matches were found between PCR‐SSO and NGS‐454 (“SSO versus 454” on Figure 1) for the 3 class II genes considered (from 71.8% for DRB1 to 89.1% for DPB1). Although both typing techniques were applied to exon 2 for these loci and were thus expected to detect identical genetic information, some groups of alleles were not detected at all by PCR‐SSO (eg, DRB1*07 and DQB1*02 alleles) and a few alleles reported with PCR‐SSO (eg, DRB1*08:02) were not confirmed by NGS‐454 **(**Supplementary Information S02). These results suggest a lack of resolution of PCR‐SSO compared with NGS‐454.

Very good matches were found between the 2 NGS techniques (“454 versus MiSeq” on Figure 1) for the 4 class II loci compared (from 88% for DPB1 to 97% for DQA1) despite the fact that they do not target the same DNA regions. NGS‐MiSeq sequences include all exons and are generally assigned to one unique HLA allele (98.7% of unambiguous typings were obtained at the third field level of resolution). By contrast, NGS‐454 sequences are only defined at exon 2 here, and are thus often ambiguously assigned to several alleles (ranging from a mean of 2.8 alleles per sequence for DPB1, to 12.3 for DQB1, see Supplementary Information S03 for a detailed list of possible alleles per NGS‐454 sequence) due to segregating sites located outside this exon. Indeed, our results show that a number of NGS‐454 alleles, some of which reach very high frequencies in the Mandenka population (Supplementary Information S02), are split through NGS‐MiSeq, namely DQB1*03:01 into DQB1*03:19, 03:01:01 and 03:01:04, and DPB1*17:01 into DPB1*17:01 and *131:01*. DQB1*03:19 (allele frequency [AF] = 0.43) is discriminated from DQB1*03:01:01 (AF = 0.07) through a segregating site located in exon 3 (codon 185: ATC → ACC); likewise, DPB1*17:01 and DPB1*131:01 (AF = 0.22 and 0.20, respectively) differ by 7 substitutions located in exon 3, plus 1 in exon 4. The very good matching scores shown in Figure 1 between NGS‐454 and NGS‐MiSeq thus correspond to genotypes with compatible allele assignments between exon 2 sequences rather than to genotypes with real allelic matches.

Finally, PCR‐SSO and NGS‐MiSeq genotypes were compared for both class I and II genes (“SSO versus MISeq” on Figure 1), revealing major differences among the loci. Fairly good concordances were achieved at the class I loci (from 78.8% for HLA‐C to 97.6% for HLA‐A), whereas very low concordances were obtained for some class II loci (18.1% for HLA‐DQB1 and 31.3% for HLA‐DPB1). A less dramatic situation was found for locus DRB1 (61% of matches), where the most frequent allele, DRB1*13:04 (frequency of almost 30% in the Mandenkalu), was detected by both techniques and was not split with NGS‐MiSeq. By contrast, at the other 2 loci the most frequent allele(s) differ(s) between PCR‐SSO and NGS‐MiSeq: with MiSeq, DQB1*03:19 was found in place of DQB1*03:01, and both DPB1*17:01 and DPB1*131:01 were found in place of DPB1*17:01. As mentioned above, these alleles differ by substitutions located outside exon 2, but these polymorphisms were unknown at the time of the PCR‐SSO typings and could thus not be reported as ambiguities. For example, the most frequent DQB1 allele found with PCR‐SSO was reported as 03:01 whereas it was ambiguously defined as 03:01 *or* 03:19 with NGS‐454 after the discovery of 03:19 in 2007,^72^ and was finally found to be 03:19 with MiSeq. This explains why NGS‐454 vs NGS‐MiSeq gave relatively high matching scores whereas PCR‐SSO vs NGS‐MiSeq gave low scores for DQB1. A similar explanation holds true for DPB1.

### 3.2 HLA molecular profile of the Mandenka population

#### 3.2.1 Allele and haplotype frequencies

HLA allele frequencies (Supplementary Information S02) and basic summary statistics at each locus (Table 3) as well as the results of global linkage disequilibrium (LD) tests between each pair of loci and the list of 2‐locus haplotypes in significant LD (Supplementary Information S04) estimated in the Mandenka population on the basis of the most precise typing technique that we used, NGS‐MiSeq, are summarized in our companion *Population Report*. 73 Relevant differences were observed between class I and class II allele frequencies, as the former are all below 20% whereas the latter always exhibit at least one allele with a frequency above 20%, that is, DRB1*13:04 (28.5%), DQA1*05:05:01 (50%), DQB1*03:19 (44.2%), DPA1*02:01:01 (46.1%) and DPB1*17:01 (21.8%, the frequency of another allele, DPB1*131:01, being very close: 18.9%). This is reflected by the contrasting diversity indexes (allelic richness, heterozygosity and *F50*) values given in Table 3, in particular between the A and B loci, on one side (*ar* ≥ 21, *H* ≥ 92%, F50 = 5), and the DQ and DP loci, on the other side (*ar* ≤16, *H* ≤ 86%, *F50* ≤ 3).

At first sight, our analyses also suggest the presence of an extended class II haplotype, DRB1*13:04~DQA1*05:05:01~DQB1*03:19~DPB1*131:01, as all allelic pairs of this putative haplotype are in significant LD (Supplementary Information S04). However, if we consider the DPA1 locus, DPA1*02:01:01 is in LD with DRB1*13:04 but not with either DQA1*05:05:01 or DQB1*03:19, and is also significantly associated with both DPB1*131:01 and DPB1*17:01. Therefore, our results merely support the existence of 3 frequent class II haplotypes, that is, DRB1*13:04~DQA1*05:05:01~DQB1*03:19, DPA1*02:01:01~DPB1*131:01 and DPA1*02:01:01~DPB1*17:01, rather than an extended haplotype across the 5 class II loci. In addition, we find no global LD between the DP loci and the other class II genes, in agreement with the existence of (a) recombination hotspot(s) between DQB1 and DPA1 near the TAP2 genes^74^, ^75^ whereas DRB1, DQA1 and DQB1 are strongly associated with each other, as are DPA1 and DPB1 (Supplementary Information S04).

#### 3.2.2 Nucleotide diversity

**Figure 2:**
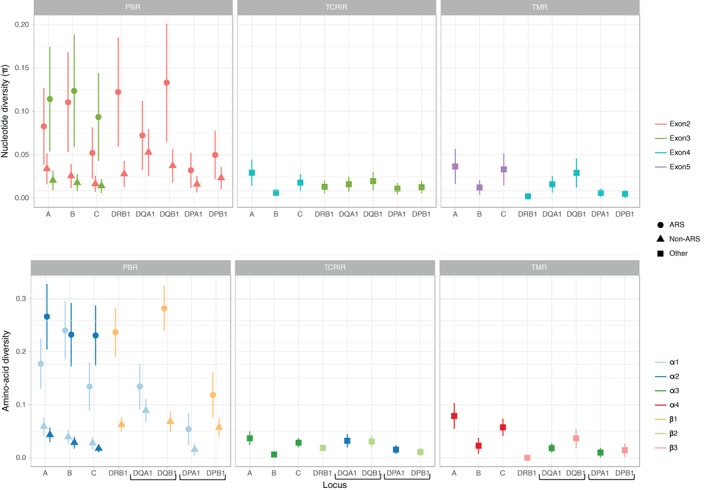
Nucleotide (top) and inferred amino acid (bottom) diversity per site (±σ) at exons encoding the peptide‐binding region (left, with a distinction between antigen‐recognition sites (ARS) and non‐antigen‐recognition sites (non‐ARS) sites); the domains interacting with CD4+ and CD8+ T‐cell receptors (middle); and the trans‐membrane region (right) of the HLA‐A, ‐B, ‐C, ‐DRB1, DQA1, DQB1 and DPB1 molecules in the Mandenka population. Brackets remind the chains forming the HLA‐DQ (DQA1 and DQB1) and HLA‐DP (DPA1 and DPB1) dimers

Figure 2 shows the nucleotide diversity per site (π ± σ) at exons 2, 3, 4 (for class I and class II) and 5 (for class I) of the 8 HLA loci (with distinction between ARS and non‐ARS codons for the peptide binding region) and the amino acid diversity per site (estimated after translating all codons into amino acids) at the corresponding α1‐α4 and β1‐β3 domains of the HLA molecules, arranged by structurally comparable elements (peptide binding region [PBR], T‐cell receptors interaction region [TCRIR] and trans‐membrane region [TMR]), in the Mandenka population. Other regions were discarded from the analysis because they were insufficiently covered by sequencing.

At both class I and class II genes, the nucleotide diversity appears to be greater at ARS than at non‐ARS codons of exons 2 (for class I and II) and 3 (for class I) encoding the PBR (top left of Figure 2). The differences between ARS and non‐ARS are even more pronounced (ie, most often significant) if we consider the amino‐acid diversity of the corresponding domains of the HLA molecules (bottom left of Figure 2), in support of an advantage of diversity at the sites involved in peptide presentation.^76^, ^77^ We also observe a lower ARS diversity at the α1 (encoded by exon 2) than at the α2 (encoded by exon 3) domain of HLA‐C and (to a lesser degree) HLA‐A, suggesting that these molecules (particularly HLA‐C) are less prone to diversifying selection at their α1 domains. By contrast, the ARS diversity is particularly high at the β1 domain (encoded by exon 2) of HLA‐DQB1 and ‐DRB1 compared with the α1 and/or β1 domains of other class II molecules. Finally, both the nucleotide and amino‐acid diversities observed within the other structural elements (TCRIR and TMR) are low (below 0.05 and 0.1, respectively), with little differences among the loci, and of the same range as those observed at non‐ARS sites within the PBR. The only locus showing such a low diversity at ARS sites is HLA‐DPA1.

### 3.3 Selective neutrality across the HLA regions

#### 3.3.1 Selective neutrality tests

We applied Tajima's test of selective neutrality on all gene regions (ie, exons and introns) sufficiently covered by sequencing of the 8 studied HLA loci **(**Supplementary Information S05). Most Tajima's *D* values are positive and sometimes significant, but only 4 regions still exhibit a significant value after correction for multiple tests (Table 4), 3 of them being neighbouring regions (in significant linkage disequilibrium, results not shown) within the DPB1 locus [DPB1 exon 2 (ARS)—DPB1 intron 2—DPB1 exon 3]. We also observe a much higher proportion of segregating sites (S) at DPB1 exon 2 (ARS) (S = 8 for 75 nucleotides, that is, 10.7%) than at DPB1 exon 3 and intron 2 (2.5% and 3.3%, respectively, Table 4).

**Table 4:** Results of Tajima's *D* and *dN/dS* selective neutrality tests for the 7 (among 86) regions rejecting significantly the null hypothesis of selective neutrality according to one or both tests (see Supplementary Information S05 for the results relative to the other regions)

Gene region	B exon 2 (ARS)	A exon3 (ARS)	B exon 3 (ARS)	DPA1 intron 1	DPB1 exon 2 (ARS)	DPB1 intron 2	DPB1 exon 3
**Size (bp)**	66	54	54	3584	75	4014	282
**S**	18	17	12	106	8	133	7
**Tajima's D**	2.4	2.9	2.3	**3.5**	**3.7**	**4.0**	**4.0**
**Adj. p‐value**	.07	.06	.08	**0**	**0**	**0**	**0**
**dN/dS**	**6.6**	**3.7**	**8.0**	‐	**dN = .07, dS = 0**	‐	.2
**Z value**	**2.91**	**3.02**	**2.29**	‐	**2.29**	‐	‐1.17

Size (bp), length of the region in base pairs; S, number of segregating sites; Adj. p‐value, p value corrected for multiple testing according to Benjamini Hochberg (fdr), *α* = .05. *Z* value is significant when outside the [‐1.96:1.96] interval. Values in bold are significant.

Because genomic regions evolving under a neutral model of molecular evolution are expected to exhibit similar proportions of non‐synonymous (*dN*) and synonymous (*dS*) nucleotide substitutions,^78^ we tested a putative deviation from 1 of the *dN*/*dS* ratio at each HLA exon of each locus (Supplementary Information S05). We found 4 regions with a *dN/dS* ratio significantly greater than 1, that is, the ARS codons of HLA‐A and ‐B exons 3 and of HLA‐B and ‐DPB1 exons 2.

#### 3.3.2 PCA of molecular diversity indices

**Figure 3:**
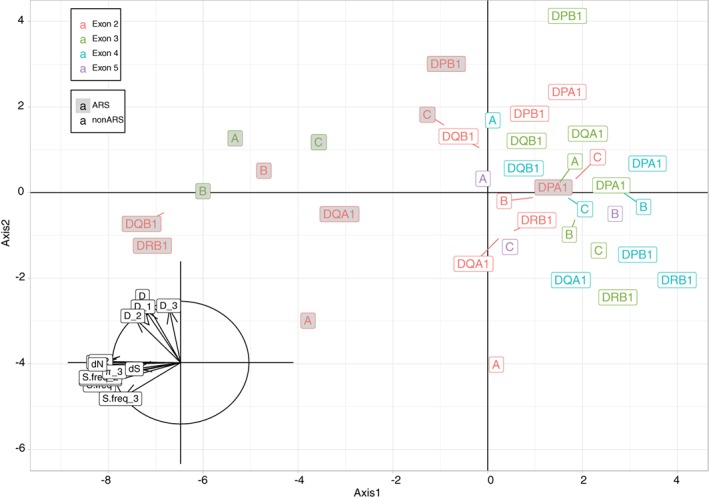
Principal component analysis (axes 1 and 2, explaining respectively 60% and 20% of the total variance) based on Tajima's *D*, nucleotide diversity π, frequency of segregating sites S.freq, number of non‐synonymous *dN* and synonymous *dS* nucleotides at exons 2, 3 and 4 of loci HLA‐A, ‐B, ‐C, ‐DRB1, ‐DQA1, ‐DQB1, ‐DPA1, ‐DPB1 and exons 5 of loci HLA‐A, ‐B, ‐C. Symbols D, D_1, D_2, D_3 represent Tajima's *D* estimated on the whole gene region and at the first, second and third nucleotide of each codon, respectively. Similarly, π, π_1, π_2 and π_3 as well as S.freq, S.freq_1, S.freq_2 and S.freq_3 represent the nucleotide diversity π and the frequency of segregating sites S estimated on the whole gene region and at nucleotide positions 1, 2 and 3 of each codon, respectively. Grey boxes correspond to ARS codons, white boxes to non‐ARS codons. The inset graph at the bottom left represents the correlations between the projections of the variables (for each pair of variables, the correlation is measured by the cosine of the angle of the 2 variable vectors) on the plan of the PCA

To better understand the results of our nucleotide diversity analyses and selective neutrality tests, we compared more in depth the molecular diversity of exons 2, 3 (split into ARS and non‐ARS codons for class I exons 2 and 3 and class II exons 2) and 4 of the 8 sequenced HLA genes and of exon 5 of HLA class I genes by including all estimated indexes related to genetic selection (ie, Tajima's *D*, nucleotide diversity *π*, frequency of segregating sites *S.freq*, number of non‐synonymous *dN* and synonymous *dS* nucleotide substitutions, computed both across the whole gene region and at the first, second (non‐degenerate) and third (degenerate) nucleotide of each codon, respectively) in a principal component analysis (PCA, Figure 3). The ratio *dN/dS* was not used in this study because both *dN* and *dS* values were already included and because HLA‐DPB1 ARS and exon 4 exhibit *dS* values of 0, therefore making *dN/dS* undetermined.

The inset graph at the bottom left of Figure 3 represents the correlations between the projections of the variables (for each pair of variables, the correlation is measured by the cosine of the angle of the 2 variable vectors) on the plan of the PCA. The first 2 principal components (PC), axes 1 and 2, explain together 80% of the total variance, and the third PC adds another 11% (see Supplementary Information S06). The first PC (60%) appears to be (inversely) correlated to the amount of molecular diversity described by *π*, *S.freq* and *dN* indexes (left side of the graph). Therefore, the left part of the graph mostly indicates non‐synonymous molecular variation (which fits with the left position of *dN*), whereas the right part indicates low molecular variability. The second PC seems to be directly correlated with Tajima's *D* values (both global and by positions). It is thus expected to discriminate regions evolving under balancing selection (top) and purifying selection (bottom). Note, however, that demographic effects may also affect *D* values,^64^, ^65^ that is, population expansion towards negative *D* values (bottom of the graph).

Interestingly, the first PC discriminates most ARS codons (except that of HLA‐DPA1), on the left, from non‐ARS codons (except those of HLA‐DQA1 and DQB1), on the right, all other codons (exons 4 and 5) falling also on the right side of the graph. This confirms that the greatest amount of HLA molecular variation is generally concentrated within the ARS, and is thus related to the peptide presentation function of the HLA molecules. Interestingly, the most extreme positions on the left are those of the ARS of DQB1, DRB1 and B exons 2 and of A exon 3. Along the second PC, most regions projected at the top of the graph deviate significantly from selective neutrality according to individual Tajima's D tests (Supplementary Information S05). However, DPB1 exon 2 regions (both ARS and non‐ARS, extreme top) show the strongest signals of balancing selection (Tajima's *D* > > 0 remaining significant after correction for multiple testing) despite their lower nucleotide diversity compared with most other exons 2 and 3. Interestingly, this axis also discriminates HLA‐A exon 2 (both ARS and non‐ARS) in its bottom part due to very low (partly negative) Tajima's *D* values (Supplementary Information S05), which may be interpreted either as purifying selection or, more likely, as population expansion (see section 4).

## 4 DISCUSSION

In this study, our objective was to provide insights into the molecular diversity and evolution of the HLA genomic region by deciphering the fine nucleotide diversity of 8 HLA loci in a well‐characterized human population. To that aim, we used 2 NGS techniques to sequence HLA genes in a West‐African population sample that we formerly typed by PCR‐SSO for several HLA loci within the frame of a wider research project investigating the genetic diversity of many independent loci of the genome in this population (see references in the Introduction). This allowed us, first, to evaluate, on the same population sample, the advantage acquired by using NGS rather than classical molecular technologies (eg, PCR‐SSO) for the characterisation of HLA population diversity and, second, to explore in detail the nucleotide variation within and among distinct gene regions of the 8 HLA loci.

### 4.1 Contribution of NGS to the assessment of HLA population diversity

Previous studies have evaluated the accuracy of different HLA‐typing methods, for example, by comparing PCR‐SSO to NGS‐454^79^ or PCR‐SSO to paired Illumina MiSeq short‐reads^80^ typings for HLA class I. The proportion of matches between PCR‐SSO and NGS‐MiSeq found by Major et al in the latter study (after applying on those data a similar way to count the matches as we did in our study, that is, by counting a match only when the 2 alleles of an individual were compatible or identical between the 2 methods, and a mismatch when at least one of the 2 alleles was discordant) is slightly different to ours (84.7%, 93.9% and 85,6% at HLA‐A, ‐B and ‐C, respectively, vs 97.6%, 91.5% and 78.8% in our study). Actually, some alleles were unknown (and thus not detectable) at the time of the PCR‐SSO studies, for example, HLA‐A*11:50Q, *01:11N* and *03:21N*, first reported between 2005 and 2009, were found with NGS‐MiSeq by Major et al^80^ but mistyped with PCR‐SSO according to the HapMap data (described in 2003^81^) used by these authors, which mainly explains why the proportion of matches is lower at HLA‐A in their study compared with ours; also, HLA‐C*07:18, first reported in 2002, was often mistyped by PCR‐SSO (in the late 1990s) as *07:01* in the Mandenka where this allele is frequent, explaining in part why the proportion of matches is lower at HLA‐C in our study compared with Major et al. This also suggests that by the time of our PCR‐SSO study, primers were used to be developed for alleles frequent in Europe and North‐America, and were thus likely to be less adapted to type African samples. Nowadays, class II alleles are also defined by taking into account nucleotide substitutions at exon 3, which explains why, by the time of the first PCR‐SSO typings, many HLA alleles had still to be discovered. Indeed, taking into account the molecular information of exon 3 did allow us to better identify the most common DQB1 allele found in the Mandenka population, DQB1*03:19, which was first defined in 2007.^72^ Also, half of the DPB1 alleles observed in this study (among which DPB1*131:01, described in 2010) were not reported before 1999, while their cumulated frequency reaches 33% in the Mandenka population, thus probably explaining the low matching score between PCR‐SSO and NGS‐MiSeq typings obtained for DPB1. By contrast, of the 21 alleles observed at the DRB1 locus, only 2 minor (ie, less frequent) alleles were unknown before 1992 (DRB1*12:10 and DRB1*14:54:01). These results confirm that we acquired a significant amount of information on HLA class II, and more particularly on DQB1 and DPB1 diversity, by improving our typing strategies. The kind of typing mismatches found in this study has to be kept in mind when using traditional typing techniques (like PCR‐SSO) or limited sequencing strategies (like single exon NGS‐454 sequencing) rather than full‐gene sequencing for the characterisation of HLA genetic profiles. This also suggests that care should always be taken in interpreting results depending on the technique used. In particular, potential ambiguities that are present at the time of the analyses should always be reported rather than “solved” by arbitrary or *ad hoc* procedures, even when using sequence‐based typings.^82^ This can be easily achieved using UNIFORMAT^83^ or alternatives like Genotype List strings^84^ or MIRING.^85^

#### 4.1.1 Gene conversion and pathogen‐driven selection (selective sweep) at HLA loci

The HLA allelic profile characterising the West African Mandenka population (the basic statistics of which are given in our companion *Population Report* 73) brings us new insights into the mechanisms that drove the evolution of this polymorphism, mostly because this population lives in a region where infectious diseases, like malaria, are highly prevalent. Surprisingly, the Mandenkalu do not exhibit a particularly high frequency of B*53:01:01 (Allele Frequency [AF] = 0.06)—the most commonly recognised HLA class I allele protective to this disease—contrary to what is observed in most West‐African regions as a putative result of resistance to *Plasmodium falciparum*.^29^ However, the most frequent HLA‐B allele observed in the Mandenkalu, B*35:01:01 (AF = 0.16), has been reported as protective to malaria in Ghana^86^ and its predicted peptide‐binding profile is similar to that of B*53:01:01,^29^ which also suggests a protective effect to this disease (this is also the case, although to a lesser extent, for the following 2 most common alleles found in this population, B*15:03:01 (AF = 0.08) and B*78:01:01 (AF = 0.08)). This indicates that distinct HLA alleles may be interchangeable in terms of protection to given pathogens.

On the other hand, we also wondered why a unique HLA class II haplotype in strong linkage disequilibrium, DRB1*13:04~DQA1*05:05:01~DQB1*03:19, was so frequent in the Mandenkalu, a large population that was not particularly prone to rapid genetic drift (see section1). Interestingly, DRB1*13:04, which was previously identified as a predominant allele in West Africa,^19^, ^87^ was proposed to originate from an allelic conversion from DRB1*11:02 based on both serological^88^ and RFLP1^87^ analyses. We investigated more in depth this hypothesis comparing the sequences of the DRB1*13:04 exons to those of the 1913 other DRB1 alleles taken from the IMGT/HLA database 3.25.0. As the allelic conversion is supposed to have happened on exon 2, we expected the 5 other exons to be identical between DRB1*13:04 and the putative recipient allele of the allelic conversion.

**Figure 4:**
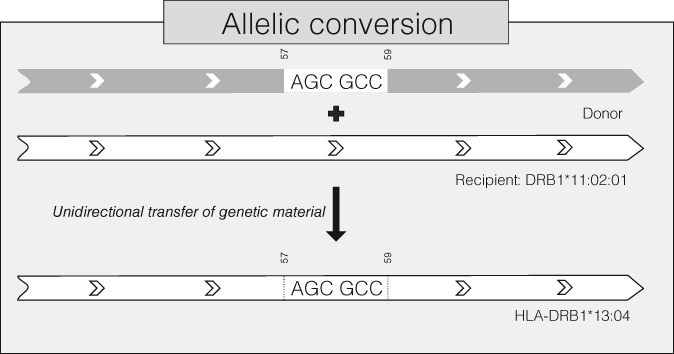
Putative mechanism of the allelic conversion mentioned in this study, which suggests an unidirectional transfer of genetic material including the « AGCGCC » pattern from a donor allele (potentially DRB1*04:05:01, DRB1*08:06 or DRB1*13:03:01 in the Mandenka) to the recipient allele DRB1*11:02:01, leading to the creation of the DRB1*13:04 allele

Of the 1913 DRB1 alleles studied (defined at the third field level of resolution in the database), 86 had differences (ranging from 4 to 22 substitutions) with DRB1*13:04 only in exon 2. The span from the first to the last substitution ranged from 30 to 241 base pairs (bp), apart from DRB1*11:02:01 for which the 5 substitutions were on a 6 bp‐long fragment, AGCGCC. Of the 1913 alleles, 195 (Supplementary Information S07) had the AGCGCC pattern, with (compared with DRB1*11:02:01/DRB1*13:04 exons 2) 32±28 conserved nucleotides before and 32±16 conserved nucleotides after the fragment (the underlying hypothesis here being that the longer the fragment, the more likely the recombination could happen), among which DRB1*08:03, which was formerly proposed as a donor for this gene conversion.^88^ However, DRB1*08:03 was not detected in the Mandenkalu and this allele is frequent in South‐East Asia, but not in Africa (at the opposite of DRB1*13:04). On the other hand, among the 195 potential donor alleles, 3 are observed in the Mandenkalu, that is, DRB1*04:05:01 (AF = 0.008), DRB1*08:06 (AF = 0.054) and DRB1*13:03:01 (AF = 0.023). We thus find very likely that a gene conversion generating DRB1*13:04 occurred (likely in West Africa) with DRB1*11:02:01 as recipient and with 1 of the 3 alleles listed above as possible donors. A scheme of the proposed allelic conversion is shown in Figure 4. Allelic conversion being a frequent event in the HLA region,^89^ this illustrates very well the progress brought by NGS: a former hypothesis of allelic conversion based on serological and RFLP typings has been refined thanks to detailed DNA sequence information.

A further challenge was to understand the very high frequency of the DRB1*13:04 allele (AF = 0.284) in the Mandenkalu, such a high allelic frequency being a rare situation at locus DRB1 in populations which have not been submitted to rapid genetic drift, like the Mandenka. To our knowledge, DRB1*13:04 has not been reported as protective for any infectious disease so far, whereas the alleles found in linkage disequilibrium at the other class II loci, namely DQA1*05:05:01 and DQB1*03:19, are potential targets of positive selection, as explained hereafter. Actually, DQB1*03:19 was only discovered in 2007, when Witter et al^72^ found that it differed from DQB1*03:01 by a single non‐synonymous substitution in the third exon (position 544 C → T), whereas exon 2 is identical. Interestingly, the combination of DQB1*03:01 and DQA1*05:01 (whose peptide‐binding region is also identical to that of DQA1*05:05) was reported by Meyer et al^90^ to be more frequent in individuals putatively immune to onchocerciasis disease, suggesting a role of these 2 alleles in the immune defence against the filarial parasitic worm *Onchocerca volvulus* (*O. volvulus)*. Onchocerciasis, or “river blindness,” is a highly prevalent disease in West Africa, including Eastern Senegal.^91^ Although it does not directly kill the infected people, it leads to severe disabilities and decreases life expectancy due to a reduced immunity. As DQB1*03:19 probably behaves like DQB1*03:01 regarding peptide presentation (their unique amino acid difference being in the β2 domain), a likely hypothesis is thus to consider DQB1*03:19 as a protective allele to *O. volvulus*, which would have led to a strong selective sweep increasing the frequency of the whole haplotype DRB1*13:04~DQA1*05:05:01~DQB1*03:19 in the Mandenkalu. The fact that this West African population lives in an area where *O. volvulus* is highly prevalent^91^ strongly supports this hypothesis. The hypothesis formerly proposed by Hill et al in 1992^87^ of recent directional selection to account for the high frequency of DRB1*13:04 in Gambia finds here a more precise explanation.

#### 4.1.2 Signatures of natural selection and demography on distinct HLA gene regions

Taken individually, several exons 2 ARS regions (B, C, DPB1, DQB1), all exons 3 ARS regions of class I (A, B, C) loci, several exons 3 of class II (DQA1, DQB1, DPB1) loci and a few exons 4 (A, DQB1) generated positive and significant Tajima's *D* values in the Mandenkalu (Supplementary Information S05), as previously found for exon2/exon 3 sequences at most HLA loci in other populations and explained by balancing selection.^13^ However, only 2 of the 49 exons included in our analyses, i.e. DPB1 exon 2 ARS and exon 3, still reached significance towards an excess of heterozygotes after correction for multiple tests (86 tests, considering all exons, introns and ARS/non‐ARS regions). Moreover, because these 2 regions are in significant linkage disequilibrium, heterozygous advantage did not necessarily affect both of them, as “associative balancing selection” may have also occurred.^92^, ^93^, ^94^ Also, *dN/dS* ratio were only significant at ARS codons of 4 regions, that is, B and DPB1 exons 2, and A and B exons 3 (note, however, that this latter result must be taken with caution as d*N*/d*S* ratio tests on molecular data were originally developed to detect selection by comparing different species and may not be adequate to infer selective pressures for samples drawn from a single population^95^). We thus find limited evidence of natural selection by means of neutrality tests applied to our data, which might be due either to a lack of statistical power or to the interplay of multiple selective forces (eg, both positive and balancing selection).

Actually, the PCA (Figure 3) based on different molecular diversity indices estimated on the 8 HLA genes (Tajima's *D*, nucleotide diversity π, frequency of segregating sites, number of non‐synonymous d*N* and synonymous d*S* nucleotide substitutions) revealed remarkable differences between distinct gene regions and loci, suggesting signatures of diverse evolutionary pressures acting across the HLA loci. In the PCA, the weight of the first PC (60% of the total variance) highlights the importance of amino acid diversity (ie, non‐synonymous substitutions) within the HLA peptide‐binding region, also revealed in Figure 2.

Interestingly, however, not all exons 2 and (for class I) exons 3 seem to be equally targeted by this kind of selection. At exon 3, the 3 class I genes exhibit, as expected, a significantly greater level of diversity at their ARS (the highest being at locus A) than at their non‐ARS codons, which is easily explained by the advantage of amino acid variation in the α2 domain of the HLA class I molecules involved (with α1) in peptide presentation. Interestingly, at ARS codons locus C is less diverse than loci A and B, and more particularly at exon 2, a plausible explanation being the main role of the corresponding α1 domain (eg, amino acid position 80) of the HLA‐C molecule as a KIR ligand,^96^, ^97^ which would weaken the advantage of amino acid diversity related to peptide binding. This hypothesis is in line with Bitarello et al 2016's^11^ observations of a lower molecular diversity of HLA‐C at the antigen recognition site (ARS) although these authors did not report detailed results on exons 2 and 3. Among the 5 class II genes, DPA1 has the less variable ARS at exon 2, suggesting a marginal contribution of this locus to peptide binding, in agreement with the low number of DPA1 alleles reported in the IMGT/HLA database, whereas B, DRB1 and DQB1 exons 2 are the most variable of all HLA loci.

To our surprise, the PCA also revealed an outlier position of locus A exon 2 (both ARS and non‐ARS codons) towards the bottom of the second PC axis, indicating reduced Tajima's *D*'s, actually a very large negative value (−0.89) at its non‐ARS codons probably explaining the negative *D* value (−0.42) found for HLA‐A exon 2 as a whole (Supplementary Information S05). Although these negative values are not significantly different from 0, they might represent a signal of demographic expansion (rather than purifying selection), as such a signal is expected in the Mandenka population based on both its known demographic history (see section 1) and other genetic studies (eg, Reference 25). This would also support the hypothesis that the evolution of the HLA‐A polymorphism (at least at the level of its exon 2 region, as suggested by the present study) is closer to neutrality and more prone to reveal demographic signals.^28^, ^29^, ^98^, ^99^ At the opposite of locus A exon 2 along the second axis of the PCA lie DPB1 exons 2 and 3. As the 2 most common alleles of this locus, DPB1*17:01 and DPB1*131:01, share an identical exon 2 (they differ at exon 3), a similar selective pressure involving an adaptive resistance to a specific pathogen (to define) would explain their high frequency in the Mandenkalu (cumulated frequency of 42%, close to that of DQB1*03:19). Actually, the DPB1 frequency distribution is much more even than that observed at other loci (as shown in Supplementary Information S02) due to high frequencies (>10%) of 4 different alleles (ie, DPB1*02:01:02 and DPB1*01:01:01, in addition to DPB1*17:01 and DPB1*131:01), explaining why DPB1 exon 2 deviates significantly from neutrality, even after correction for multiple tests, towards an excess of heterozygotes. At this locus, the significant excess of heterozygotes also observed at exon 3 would then be due to linkage disequilibrium with exon 2. This result contrasts with previous studies where DPB1 was found to exhibit a more “L‐shaped” (ie, neutral‐like) distribution than the other loci.^13^, ^100^ However, as DQA1, DQB1 and DRB1 (in linkage disequilibrium) have possibly been submitted to a strong selective sweep due to resistance to *O. volvulus* in this population, the resulting loss of diversity at these genes may have been compensated by a greater (and significant) heterozygous advantage at DPB1 (not affected by the selective sweep) conferring protection to other pathogens also present in the environment. Interestingly, this explanation fits with the model of *joint divergent asymmetric selection* recently proposed by Buhler et al in 2016^37^ to explain the sharp differences of heterozygosity sometimes found among distinct class I loci. The putative existence of such similar mechanisms at both class I and class II genes makes this model appear even more robust and consistent in the evolution of the HLA region.

## 5 CONCLUDING REMARKS

The recent development of high‐throughput sequencing technologies applied to HLA can be considered as a major upheaval: as shown in the present study, not only the typing errors dropped, but the deciphering of the fine nucleotide diversity of different HLA gene regions opens new ways to explore the evolution of this exceptional polymorphism and to understand better the mechanisms of our immune defences. With the generalization of these methodologies, the number of HLA named alleles is expected to explode in the next years, notably at the fourth field level of resolution describing the variability of introns and other untranslated regions. While this will probably represent a turning point in the way the HLA polymorphism is reported—a real challenge for the nomenclature committee and a delicate shift for HLA researchers and clinicians in histocompatibility—it will also certainly catalyse our understanding of the hidden face of the HLA genomic region: how such crucial genes are regulated. In the meanwhile, we can already say that a new and promising period for researchers in HLA molecular population genetics just started.

## Supporting information


**Supplementary Information S01** Additional information on population sampling, DNA extraction, HLA typings and sequencing methods

**Supplementary Information S02** Distribution of HLA allele or sequence frequencies and basic statistics based on PCR‐SSO (left), NGS‐454 (middle) and NGS‐MiSeq (right) typings in the Mandenka population

**Supplementary Information S03** NGS‐454 assigned sequence numbers with corresponding alleles

**Supplementary Information S04** Global linkage disequilibrium between the 8 HLA loci

**Supplementary Information S05** Statistics computed for the assessment of selective neutrality in each gene region

**Supplementary Information S06** Principal Component Analysis, axes 1 and 3

**Supplementary Information S07** Potential donors for the DRB1*13:04 allelic conversion
