# Supplementary material for: Deciphering the fine nucleotide diversity of full HLA class I and class II genes in a well‐documented population from sub‐Saharan Africa
Source: HLA. 2017 Dec 25;91(1):36–51. doi: 10.1111/tan.13180 (PMC5767763; doi:10.1111/tan.13180)
Supplement: Supplementary file 1 — Supplementary Information S01 Additional information on population sampling, DNA extraction, HLA typings and sequencing methods [file TAN-91-36-s001.pdf]

Supplementary Information S01

Additional information on population sampling, DNA extraction, HLA typings and sequencing methods.

Population sampling and DNA extraction

The Mandenka individuals included in this study come from five villages (Batranke, Bantata, Baraboye, Lakanta and Soucouta) from the Niokholo region of the Bandafassi district (Eastern Senegal, West Africa), at about 30 km from the city of Kédougou (**Figure S1**).

Blood samples were collected in Vacutainer sterile tubes, and a half volume of RPMI medium supplemented with 10 % FCS and heparin (1 % final concentration) was added. DNA was extracted from buffy coats by the high salt procedure as described in Tiercy *et al* 1989[1]. Optic Density was measured using a nanodrop 2000 and DNA was then frozen at -20°C until used. DNA samples were then resuspended to have a concentration of 30 ng.µl-1 for further use.

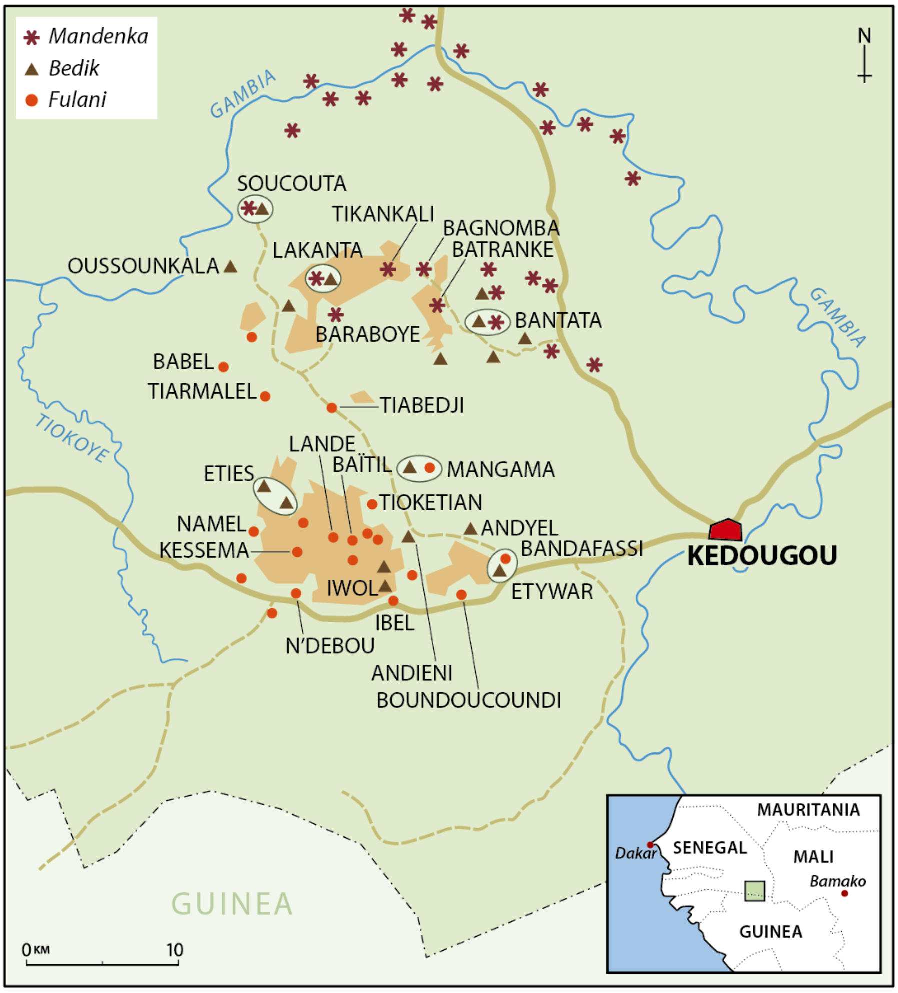

***Figure S1**: Sampling location[2], near the Kédougou city in Eastern Senegal (Niokholo region, Bandafassi district). The map shows the close geographical location between Bedik, Fulani and Mandenka villages.*

HLA typings

PCR-SSO typings of class I and II genes

HLA class II (DRB1, DQB1 and DPB1) typings were performed by locus- and group-specific PCR amplification, followed by direct hybridization with sequence-specific oligonucleotide (SSO) probes on nylon filters. HLA-DRB1 and -DQB1 SSO probes were designed[3,4] in order to discriminate the alleles assigned by the HLA Nomenclature Committee in 1991.

For HLA class I (A, B, C) typings, samples were first tested in 1996 by direct PCR-SSO hybridization, using locally designed probes[5,6,7]. In 2001 new HLA-A and -B typings were performed using the reverse PCR-SSO hybridization protocol of the 13th International Histocompatibility and Immunogenetics Workshop (IHIW, the reverse line strip system having been specifically developed for the IHIW Anthropology component) that comprised 139 probes able to discriminate 341 alleles.

NGS-454 pyrosequencing of class II genes' exon 2

Four HLA Class II genes (DRB1, DQA1, DQB1, DPB1) were amplified and sequenced using this method. For each gene, 245 samples (+12 control H_2O) were amplified. A total of 46 replicates (19%) were used to confirm the sequence.

Tagging and multiplexing methods designed by Galan *et al*[8] for Roche NGS-454 sequencing were used. For this high-throughput sequencing, adaptors are required for the emPCR and 454 GS-FLX pyrosquencing using Lib-L Titanium Series reagents. Thus, the primers used (**Table S1**) were modified by adding a 30 bp Titanium adaptor to the 5'-end (CCATCTCATCCCTGCGTGTCTCCGACTCAG) and a short sequence of 7-mer oligonucleotidic tag. A total of 32 forward and 21 reverse tags were designed in order to allow the generation of 182 unique combinations of forward and reverse primers and to sequence as many individually tagged amplicons at the same time. Three sequencing runs were performed.

Locus	Direction	Primer
DRB1	Forward	Adaptor + tag + CCGGATCCTTCGTGTCCCCACAGCACG
	Reverse	Adaptor + tag + CCGAATTCCGCTGCACTGTGAAGCTCTC
DQA1	Forward	Adaptor + tag + GTTTCTTYCATCATTTTGTGTATTAAGGT
	Reverse	Adaptor + tag + CGGTAGAGTTGTAGCGTTTA
DQB1	Forward	Adaptor + tag + AGGATCCCCGCAGAGGATTTCGTGTACCA
	Reverse	Adaptor + tag + TCCTGCAGGACGCTCACCTCTCCGCTGCA
DPB1	Forward	Adaptor + tag + GCTGCAGGAGAGTGGCGCCTCCGCTCAT
	Reverse	Adaptor + tag + CGGATCCGGCCCAAAGCCCTCACTC

Table S1*: Forward and reverse PCR primers used for the pyrosequencing of the four HLA class II exons 2.*

Due to primer design, exon 2 was sequenced from position 5 to position 245 for DRB1, and from 17 to 258 for DQB1; for DQA1 the sequences encompass the last 50bp of intron 1 (7 different

haplotypes) to position 217 (lacking the last 32pb of the exon); for DPB1, the sequences encompass the last 19 bp of intron 1, the complete exon 2 and the first 2 bp of intron 2 (but no variability was observed in the intronic regions).

PCR preparations were performed in 96-well plates, under a sterile hood to avoid contamination by other DNA. For every plate, a well was filled with water and PCR mix to check the absence of contamination after amplification. The fusion-primers were diluted in de-ionized contamination-free water in order to have a concentration of 0,5 µM of each primer. Amplifications were made in a 10µl reaction volume containing 5 µl of QIAGEN Multiplex Kit Buffer (*QIAGEN*), 2 µl of de-ionized DNA- and RNA-free water, 1 µl of each of the diluted primers and 1 µl of DNA. The thermocycling conditions used for the amplification began with 1 cycle of 15 min at 95°C followed by 40 cycles of 20 sec at 95°C (denaturation step), 45 sec at 55°C (annealing step) and 60 sec at 72°C (extension). A last step at 72°C was done at the end during 10 minutes.

After amplification, PCR products were checked on a 2% agarose gel using a loading dye (*Promega*) or directly on a 2% E-gel (E-gel 96 2% with SYBR safe, *Invitrogen*). A total of 4µl (more for samples with low amplification signals) of each PCR product of the entire plate were then collected in a single tube. Plates were mixed two by two in singles tubes. To ensure the same amplification between the tubes, amplifications were tested on a 2% agarose gel. Finally, the tubes were mixed together, the quantity of each depending on their amplification signals. To control that the amplification between the 4 genes was the same, products of the 4 tubes were run on a 2% agarose gel. The final 4 tubes were sent to *Beckman Coulter Genomics* to be sequenced.

Sequences were delivered as Fasta and FastQ files. Reads were filtered using Mothur[9] with a minimal PhredScore of 30. These data were explored using SESAME Barcode[10]. SESAME software processes the DNA sequences obtained through next-generation sequencing and allows the identification of multiplexed samples by the tags and a reference sequence.

NGS- MiSeq sequencing of full class I and class II genes

Two complementary techniques were used to sequence the full HLA genes at both the Geneva Hospital and Stanford University.

Geneva Hospital

We used the Holotype HLA X2 kit (*Omixon Biocomputing Ltd, Budapest, Hungary*) in combination with the Illumina MiSeq platform to type 58 individuals for 7 HLA loci (A, B, C, DRB1, DQA1, DQB1 and DPB1). Long-range PCR amplification was done for each locus. HLA-A, B, C, DQA1 and DQB1 were amplified over their entire length, from 5'UTR to 3'UTR (DQB1 needed 2 different amplifications). HLA-DRB1 was amplified from intron 1 to intron 4 and HLA-DPB1 from intron 1 to 3'UTR. The primers' mixes were provided by Omixon (*Holotype HLA X2 Kit*). The dNTP (10mM), the LongRange PCR Buffer (10x) and the LongRange PCR Enzyme Mix (*LongRange PCR kit, QIAGEN*) were used for the amplification. The amplicon sizes were verified through 2% agarose gel electrophoresis. The amplicons were quantified by qPCR, using the Quantifluor dsDNA System (*Promega*). The samples were then diluted to reach approximately the same final concentration and all the loci of each individual were pooled. The library preparation was done using the Holotype HLA X2 kit (*Omixon*), which provides buffers and enzymes for the whole process. The amplicons generated by the long range PCR were first enzymatically fragmented. The shared ends produced by this enzyme were repaired and the adaptors were ligated to the ends of the fragments. A final pool was created,

containing fragments of every locus for all the individuals. A clean-up and concentration step was performed using AMPure XP Beads (*Beckman Coulter*). A size selection of the pool was done using a Pippin Prep (*Sage Science*) in order to select a range of DNA fragment sizes. The library was finally quantified by qPCR, using the reagents from the Library Quantification Kit – Illumina/universal (*KAPA biosystems*). The denatured library was loaded at 9pM in the MiSeq. The FASTQ files generated by the MiSeq were processed by the software HLA Twin v1.1.1 (*Omixon*).

Stanford University

A total of 65 DNA samples were typed for 8 HLA loci (A, B, C, DPA1, DPB1, DQA1, DQB1, DRB1) using a NGS typing method developed by Sirona Genomics (*Immucor, Inc, Norcross, GA, USA*) and performed following the manufacturer's instructions. Long range PCR amplified the entire genes of all class I loci (5'UTR to 3'UTR) and key regions of class II loci: DPA1: 5'UTR to intron 4; DPB1: intron 1 to intron 4; DQA1: 5'UTR to intron 4; DQB1: 5'UTR to intron 5. The PCR amplification reaction contained 100 ng of genomic DNA, Sirona PCR master mix containing enzymes and primers specific for each HLA locus. The thermal cycling parameters for all genes were: initial denaturation 94°C/2 min, followed by 35 cycles at 94°C/30 sec, 60°C/30 sec, 66°C/7 min 30 sec, followed by a final extension step at 66°C for 10 min. PCR were performed using Veriti Thermal Cyclers (*Applied Biosystems/Thermo Fisher Scientific, Waltham, MA, USA*). PCR products were quantified using a PicoGreen assay (*Invitrogen/Thermo Fisher Scientific, Waltham, MA, USA*) with a Victor X plate reader (*Perkin Elmer, Waltham, MA, USA*). PCR products for all genes were pooled in optimal molar amounts, and purified using Agencourt AMPure XP beads (*Beckman Coulter, Fullerton, CA*). Barcoded sample libraries were prepared by enzymatic cleavage into 300-500 bp fragments, purification using Agencourt AMPure beads, followed by enzymatic end repair to remove dNTPs overhangs, incorporation of deoxynucleotide dAMP to blunt ended 3' ends, followed by ligation of a unique index 'barcode' adaptor to each pooled sample. All adaptor ligated samples were pooled into a single tube, purified using Agencourt AMPure XP beads and DNA fragments were size selected for 400-500 bp fragments using the Blue Pippin system (*Sage Science, Inc, Beverly, MA, USA*). The eluted sample was enriched by a short PCR cycle, quality checked using the Agilent 2200 TapeStation instrument (*Agilent Technologies, Inc, Santa Clara, CA, USA*) and quantity measured using the KAPA library universal quantification kit (*Kapa Biosystems, Inc, MA, USA*) with the 3130 Genetic Analyzer (*Applied Biosystems/Thermo Fisher Scientific, Waltham, MA, USA*). The sample was denatured with sodium hydroxide, and sequenced at a final concentration of 12 pM on the Illumina MiSeq instrument using 300 cycle paired-end V2 kits (*Illumina, Inc, San Diego, CA, USA*).

HLA alleles were assigned using the Sirona Genomics NGS alignment software, which uses two complementary informatics strategies to analyse to make genotyping calls. The first strategy utilizes Expectation Maximization to rank computed allele candidates based on mapping metrics. Coverage is calculated from competitive alignment of paired-end NGS reads with all HLA reference sequences in the IMGT/HLA database v3.22 [11] (*http://www.ebi.ac.uk/imgt/hla*) and reference sequences generated 'internally' by Sirona/Immucor. The second strategy utilizes a dynamic phasing algorithm to assemble reads and construct one or two phased consensus sequences by *de novo* assembly of mapped paired-end sequences. These consensus sequences are then aligned to the HLA allele database to find the best fit.

References

1. Tiercy JM, Gorski J, Bétuel H, et al. DNA typing of DRw6 subtypes: correlation with DRB1 and DRB3 allelic sequences by hybridization with oligonucleotide probes. *Hum Immunol*. 1989;24(1):1-14.

2. Blanc M, Sanchez-Mazas A, Van Blyenburgh NH, Sevin A, Pinson G, Langaney A. Interethnic Genetic Differentiation: GM Polymorphism in Eastern Senegal. *Am J Hum Genetb*. 1990;46:383--392.

3. Tiercy JM, Sanchez-Mazas A, Excoffier L, et al. HLA-DR Polymorphism in a Senegalese Mandenka Population: DNA Oligotyping and Population Genetics of DRB I Specificities. *Am J Hum Genet*. 1992;51:592--608.

4. Morel C, Zwahlen F, Jeannet M, Mach B, Tiercy JM. Complete analysis of HLA-DQB1 polymorphism and DR-DQ linkage disequilibrium by oligonucleotide typing. *Hum Immunol*. 1990;29(1):64-77.

5. Andrien M, Tiercy JM, Defleur V, et al. HLA-B locus DNA typing: detection of B*7801 and seven additional alleles by BW6-specific exon 2 amplification. *Tissue Antigens*. 1993;42(5):480-487.

6. Tiercy JM, Djavad N, Rufer N, Speiser DE, Jeannet M, Roosnek E. Oligotyping of HLA-A2, -A3, and -B44 subtypes. Detection of subtype incompatibilities between patients and their serologically matched unrelated bone marrow donors. *Hum Immunol*. 1994;41(3):207-215.

7. Grundschober C, Rufer N, Sanchez-Mazas A, et al. Molecular characterization of HLA-C incompatibilities in HLA-ABDR-matched unrelated bone marrow donor-recipient pairs. *Tissue Antigens*. 1997;49(6):612-623. doi:10.1111/j.1399-0039.1997.tb02809.x.

8. Galan M, Guivier E, Caraux G, Charbonnel N, Cosson J-F. A 454 multiplex sequencing method for rapid and reliable genotyping of highly polymorphic genes in large-scale studies. *BMC Genomics*. 2010;11(1):296. doi:10.1186/1471-2164-11-296.

9. Schloss PD, Westcott SL, Ryabin T, et al. Introducing mothur: Open-Source, Platform-Independent, Community-Supported Software for Describing and Comparing Microbial Communities. *Appl Environ Microbiol*. 2009;75(23):7537-7541. doi:10.1128/AEM.01541-09.

10. Meglecz E, Piry S, Desmarais E, et al. SESAME (SEquence Sorter & AMplicon Explorer): genotyping based on high-throughput multiplex amplicon sequencing. *Bioinformatics*. 2011;27(2):277-278. doi:10.1093/bioinformatics/btq641.

11. Robinson J, Halliwell JA, Hayhurst JD, Flicek P, Parham P, Marsh SGE. The IPD and IMGT/HLA database: allele variant databases. *Nucleic Acids Res*. 2015;43(D1):D423-D431. doi:10.1093/nar/gku1161.
