# Supplementary material for: Deciphering the fine nucleotide diversity of full HLA class I and class II genes in a well‐documented population from sub‐Saharan Africa
Source: HLA. 2017 Dec 25;91(1):36–51. doi: 10.1111/tan.13180 (PMC5767763; doi:10.1111/tan.13180)
Supplement: Supplementary file 2 — Supplementary Information S02 Distribution of HLA allele or sequence frequencies and basic statistics based on PCR‐SSO (left), NGS‐454 (middle) and NGS‐MiSeq (right) typings in the Mandenka population [file TAN-91-36-s005.pdf]

Supplementary Information S02

Distribution of HLA allele or sequence frequencies and basic statistics based on PCR-SSO (left), NGS-454 (middle) and NGS-MiSeq (right) typings in the Mandenka population

All frequencies and statistics have been estimated using the Gene[RATE] computer tools (*http://hla-net.eu/tools/*).
When a given locus has not been typed by a given technique, the corresponding column has been left empty.
For NGS-454, sequence numbers correspond to the lists of possible alleles given in **Supplementary Information S03**.

PCR−SSO

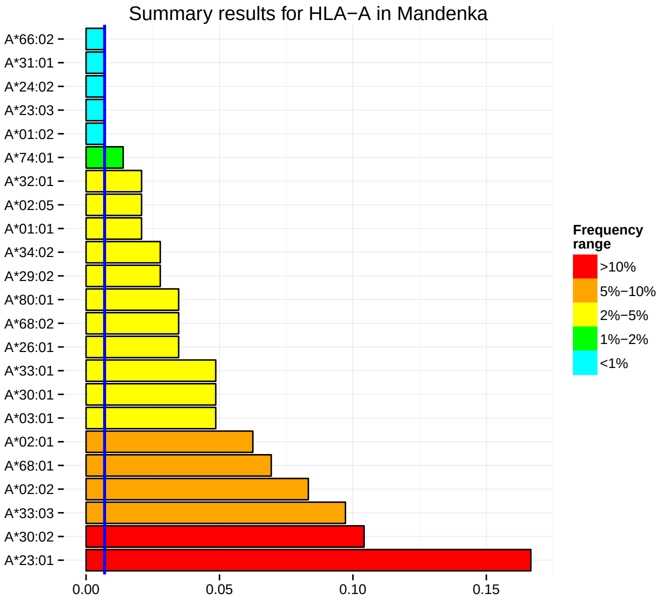

Sample size: 72

Estimated heterozygosity: 0.9223

Hardy−Weinberg equilibrium test p−value: 0.7857777

Ewens−Watterson−Slatkin neutrality test: FDR p.adj min: 0.007 max: 0.382

Follows the complete list of alleles, including those with lower and null frequencies not shown before.

NGS−454

NGS−MiSeq

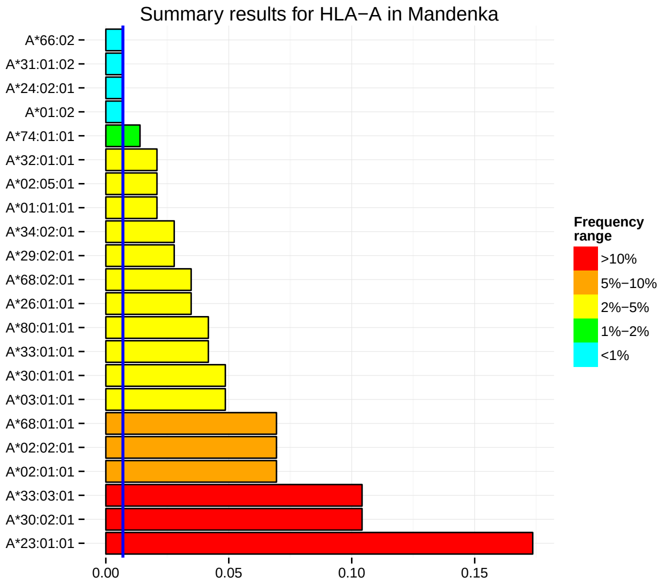

Sample size: 72

Estimated heterozygosity: 0.9199

Hardy−Weinberg equilibrium test p−value: 0.3310911

Ewens−Watterson−Slatkin neutrality test: FDR p.adj min: 0.005 max: 0.452

Follows the complete list of alleles, including those with lower and null frequencies not shown before.

PCR−SSO

	Allele or Haplotype Name	Frequency Estimate
1	A*23:03	0.0069
2	A*66:02	0.0069
3	A*01:02	0.0069
4	A*31:01	0.0069
5	A*24:02	0.0069
6	A*74:01	0.0139
7	A*01:01	0.0208
8	A*32:01	0.0208
9	A*02:05	0.0208
10	A*29:02	0.0278
11	A*34:02	0.0278
12	A*68:02	0.0347
13	A*80:01	0.0347
14	A*26:01	0.0347
15	A*30:01	0.0486
16	A*33:01	0.0486
17	A*03:01	0.0486
18	A*02:01	0.0625
19	A*68:01	0.0694
20	A*02:02	0.0833
21	A*33:03	0.0972
22	A*30:02	0.1042
23	A*23:01	0.1667

NGS−454

NGS−MiSeq

	Allele or Haplotype Name	Frequency Estimate
1	A*66:02	0.0069
2	A*01:02	0.0069
3	A*31:01:02	0.0069
4	A*24:02:01	0.0069
5	A*74:01:01	0.0139
6	A*01:01:01	0.0208
7	A*32:01:01	0.0208
8	A*02:05:01	0.0208
9	A*29:02:01	0.0278
10	A*34:02:01	0.0278
11	A*68:02:01	0.0347
12	A*26:01:01	0.0347
13	A*33:01:01	0.0417
14	A*80:01:01	0.0417
15	A*30:01:01	0.0486
16	A*03:01:01	0.0486
17	A*68:01:01	0.0694
18	A*02:01:01	0.0694
19	A*02:02:01	0.0694
20	A*30:02:01	0.1042
21	A*33:03:01	0.1042
22	A*23:01:01	0.1736

PCR−SSO

NGS−454

NGS−MiSeq

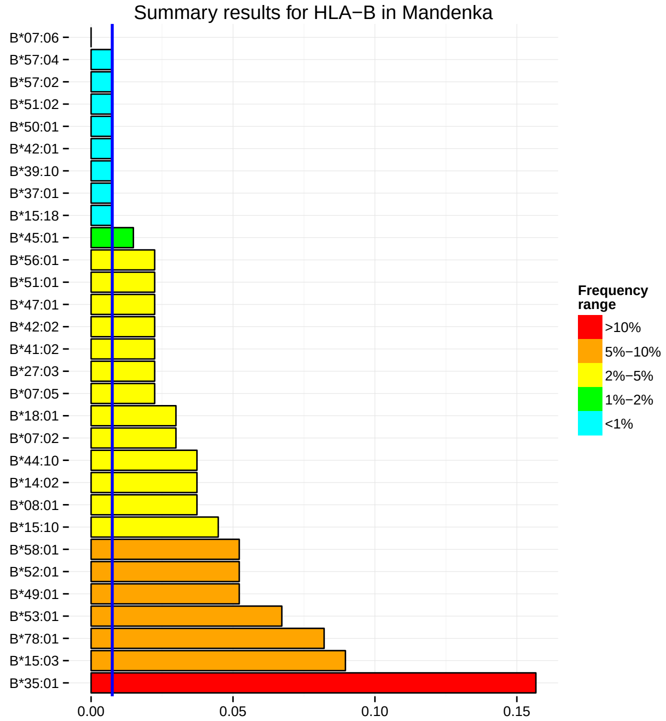

Sample size: 67
Estimated heterozygosity: 0.9358
Hardy−Weinberg equilibrium test p−value: 1
Ewens−Watterson−Slatkin neutrality test: FDR p.adj min: 0.008 max: 0.562

Follows the complete list of alleles, including those with lower
and null frequencies not shown before.

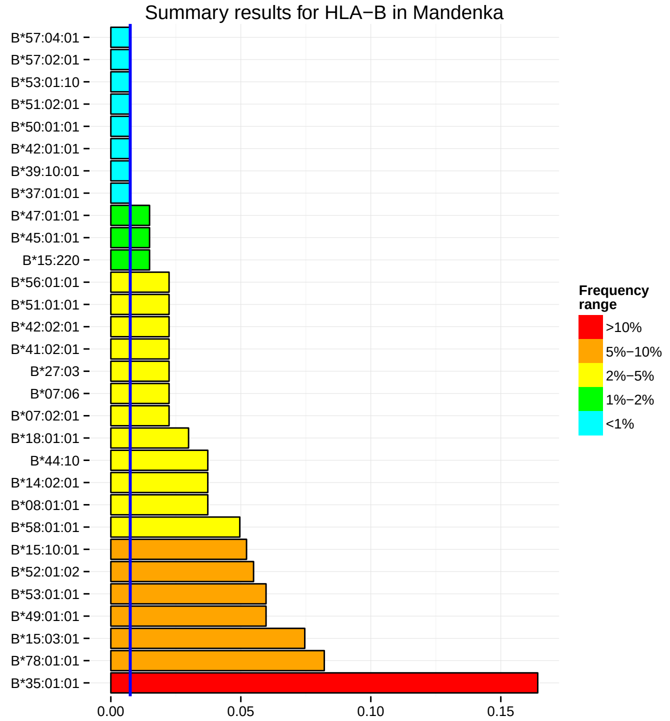

Sample size: 67
Estimated heterozygosity: 0.9357
Hardy−Weinberg equilibrium test p−value: 0.5237272
Ewens−Watterson−Slatkin neutrality test: FDR p.adj min: 0.013 max: 0.684

Follows the complete list of alleles, including those with lower
and null frequencies not shown before.

PCR−SSO

	Allele or Haplotype Name	Frequency Estimate
1	B*07:06	0.0000
2	B*15:18	0.0075
3	B*37:01	0.0075
4	B*39:10	0.0075
5	B*50:01	0.0075
6	B*57:02	0.0075
7	B*42:01	0.0075
8	B*51:02	0.0075
9	B*57:04	0.0075
10	B*45:01	0.0149
11	B*07:05	0.0224
12	B*41:02	0.0224
13	B*42:02	0.0224
14	B*47:01	0.0224
15	B*51:01	0.0224
16	B*56:01	0.0224
17	B*27:03	0.0224
18	B*07:02	0.0299
19	B*18:01	0.0299
20	B*14:02	0.0373
21	B*44:10	0.0373
22	B*08:01	0.0373
23	B*15:10	0.0448
24	B*49:01	0.0522
25	B*58:01	0.0522
26	B*52:01	0.0522
27	B*53:01	0.0672
28	B*78:01	0.0821
29	B*15:03	0.0896
30	B*35:01	0.1567

NGS−454

NGS−MiSeq

	Allele or Haplotype Name	Frequency Estimate
1	B*37:01:01	0.0075
2	B*39:10:01	0.0075
3	B*50:01:01	0.0075
4	B*57:02:01	0.0075
5	B*53:01:10	0.0075
6	B*42:01:01	0.0075
7	B*51:02:01	0.0075
8	B*57:04:01	0.0075
9	B*15:220	0.0149
10	B*45:01:01	0.0149
11	B*47:01:01	0.0149
12	B*07:06	0.0224
13	B*41:02:01	0.0224
14	B*42:02:01	0.0224
15	B*07:02:01	0.0224
16	B*51:01:01	0.0224
17	B*56:01:01	0.0224
18	B*27:03	0.0224
19	B*18:01:01	0.0299
20	B*14:02:01	0.0373
21	B*44:10	0.0373
22	B*08:01:01	0.0373
23	B*58:01:01	0.0496
24	B*15:10:01	0.0522
25	B*52:01:02	0.0549
26	B*53:01:01	0.0597
27	B*49:01:01	0.0597
28	B*15:03:01	0.0746
29	B*78:01:01	0.0821
30	B*35:01:01	0.1642

PCR−SSO

NGS−454

NGS−MiSeq

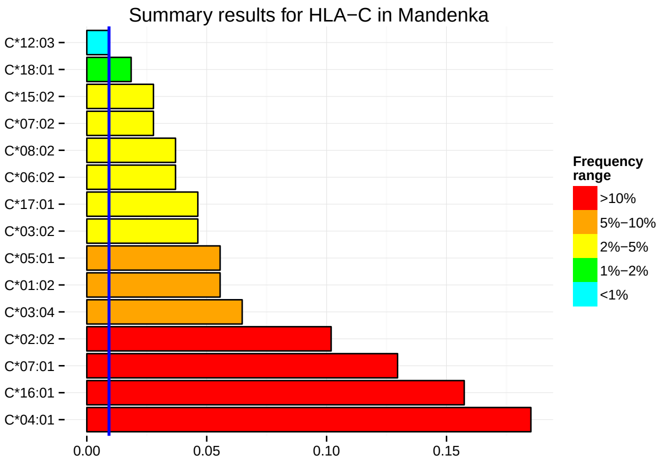

Sample size: 54
Estimated heterozygosity: 0.8944
Hardy−Weinberg equilibrium test p−value: 0.2116745
Ewens−Watterson−Slatkin neutrality test: FDR p.adj min: 0.003 max: 0.571

Follows the complete list of alleles, including those with lower
and null frequencies not shown before.

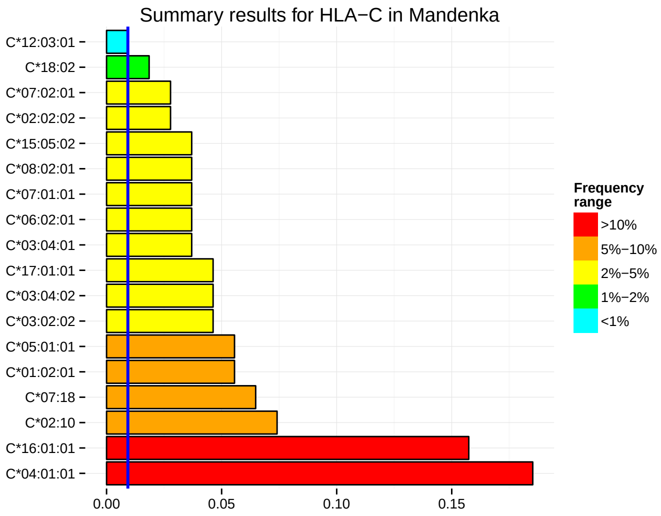

Sample size: 54
Estimated heterozygosity: 0.9098
Hardy−Weinberg equilibrium test p−value: 0.044439
Ewens−Watterson−Slatkin neutrality test: FDR p.adj min: 0.002 max: 0.272

Follows the complete list of alleles, including those with lower
and null frequencies not shown before.

PCR−SSO

	Allele or Haplotype Name	Frequency Estimate
1	C*12:03	0.0093
2	C*18:01	0.0185
3	C*15:02	0.0278
4	C*07:02	0.0278
5	C*08:02	0.0370
6	C*06:02	0.0370
7	C*17:01	0.0463
8	C*03:02	0.0463
9	C*01:02	0.0556
10	C*05:01	0.0556
11	C*03:04	0.0648
12	C*02:02	0.1019
13	C*07:01	0.1296
14	C*16:01	0.1574
15	C*04:01	0.1852

NGS−454

NGS−MiSeq

	Allele or Haplotype Name	Frequency Estimate
1	C*12:03:01	0.0093
2	C*18:02	0.0185
3	C*02:02:02	0.0278
4	C*07:02:01	0.0278
5	C*08:02:01	0.0370
6	C*15:05:02	0.0370
7	C*03:04:01	0.0370
8	C*06:02:01	0.0370
9	C*07:01:01	0.0370
10	C*17:01:01	0.0463
11	C*03:02:02	0.0463
12	C*03:04:02	0.0463
13	C*01:02:01	0.0556
14	C*05:01:01	0.0556
15	C*07:18	0.0648
16	C*02:10	0.0741
17	C*16:01:01	0.1574
18	C*04:01:01	0.1852

PCR−SSO

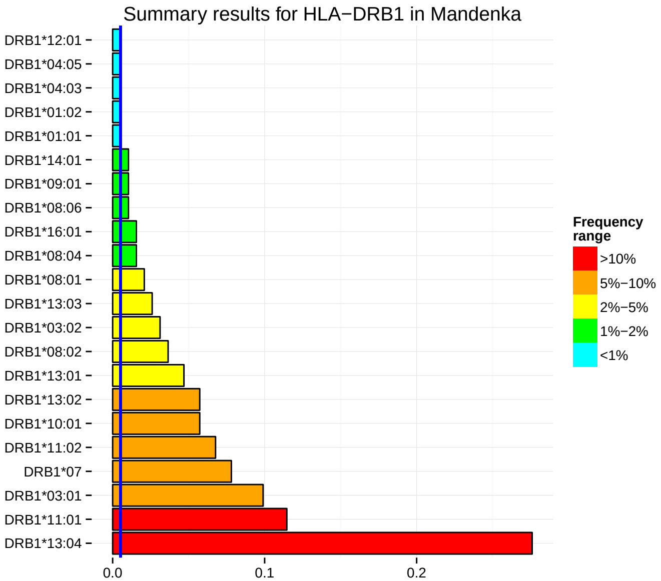

Sample size: 96
Estimated heterozygosity: 0.8771
Hardy−Weinberg equilibrium test p−value: 0.1631775
Ewens−Watterson−Slatkin neutrality test: FDR p.adj min: 0.213 max: 0.736

Follows the complete list of alleles, including those with lower
and null frequencies not shown before.

NGS−454

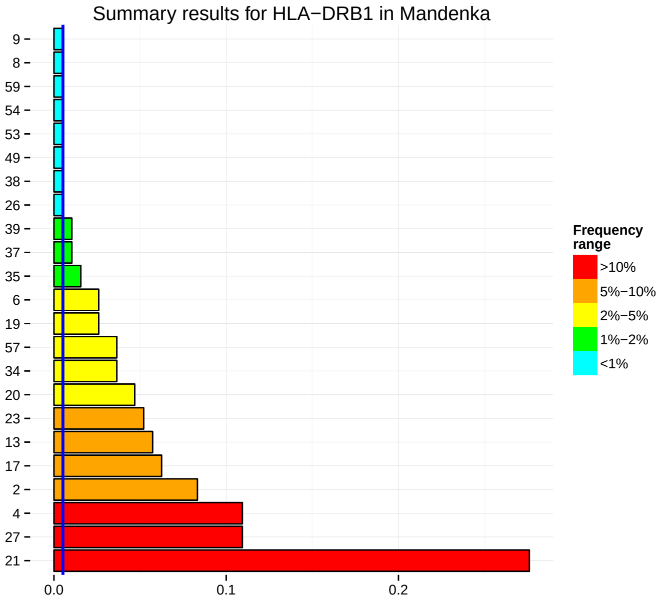

Sample size: 96
Estimated heterozygosity: 0.8762
Hardy−Weinberg equilibrium test p−value: 0.8979362
Ewens−Watterson−Slatkin neutrality test: FDR p.adj min: 0.317 max: 0.673

Follows the complete list of alleles, including those with lower
and null frequencies not shown before.

NGS−MiSeq

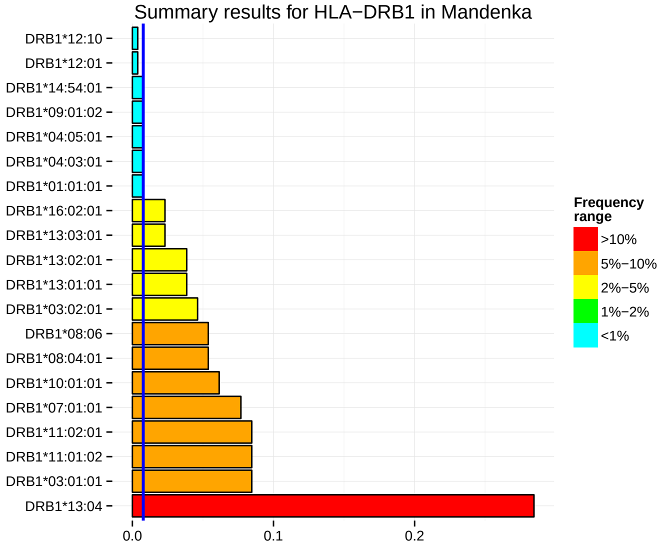

Sample size: 65
Estimated heterozygosity: 0.8756
Hardy−Weinberg equilibrium test p−value: 0.08938847
Ewens−Watterson−Slatkin neutrality test: FDR p.adj min: 0.050 max: 0.671

Follows the complete list of alleles, including those with lower
and null frequencies not shown before.

PCR−SSO

	Allele or Haplotype Name	Frequency Estimate
1	DRB1*01:02	0.0052
2	DRB1*04:05	0.0052
3	DRB1*04:03	0.0052
4	DRB1*01:01	0.0052
5	DRB1*12:01	0.0052
6	DRB1*09:01	0.0104
7	DRB1*08:06	0.0104
8	DRB1*14:01	0.0104
9	DRB1*16:01	0.0156
10	DRB1*08:04	0.0156
11	DRB1*08:01	0.0208
12	DRB1*13:03	0.0260
13	DRB1*03:02	0.0312
14	DRB1*08:02	0.0365
15	DRB1*13:01	0.0469
16	DRB1*13:02	0.0573
17	DRB1*10:01	0.0573
18	DRB1*11:02	0.0677
19	DRB1*07	0.0781
20	DRB1*03:01	0.0990
21	DRB1*11:01	0.1146
22	DRB1*13:04	0.2760

NGS−454

	Allele or Haplotype Name	Frequency Estimate
1	8	0.0052
2	26	0.0052
3	9	0.0052
4	38	0.0052
5	49	0.0052
6	53	0.0052
7	54	0.0052
8	59	0.0052
9	37	0.0104
10	39	0.0104
11	35	0.0156
12	6	0.0260
13	19	0.0260
14	34	0.0365
15	57	0.0365
16	20	0.0469
17	23	0.0521
18	13	0.0573
19	17	0.0625
20	2	0.0833
21	4	0.1094
22	27	0.1094
23	21	0.2760

NGS−MiSeq

	Allele or Haplotype Name	Frequency Estimate
1	DRB1*12:10	0.0038
2	DRB1*12:01	0.0038
3	DRB1*01:01:01	0.0077
4	DRB1*04:05:01	0.0077
5	DRB1*04:03:01	0.0077
6	DRB1*09:01:02	0.0077
7	DRB1*14:54:01	0.0077
8	DRB1*16:02:01	0.0231
9	DRB1*13:03:01	0.0231
10	DRB1*13:02:01	0.0385
11	DRB1*13:01:01	0.0385
12	DRB1*03:02:01	0.0462
13	DRB1*08:04:01	0.0538
14	DRB1*08:06	0.0538
15	DRB1*10:01:01	0.0615
16	DRB1*07:01:01	0.0769
17	DRB1*03:01:01	0.0846
18	DRB1*11:02:01	0.0846
19	DRB1*11:01:02	0.0846
20	DRB1*13:04	0.2846

PCR−SSO

NGS−454

NGS−MiSeq

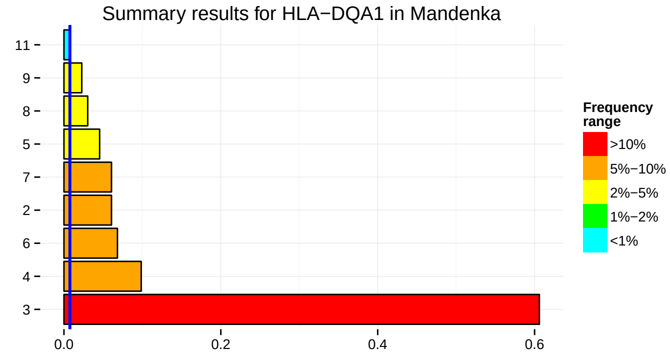

Sample size: 66
Estimated heterozygosity: 0.6074
Hardy−Weinberg equilibrium test p−value: 1
Ewens−Watterson−Slatkin neutrality test: FDR p.adj min: 0.521 max: 0.732

Follows the complete list of alleles, including those with lower
and null frequencies not shown before.

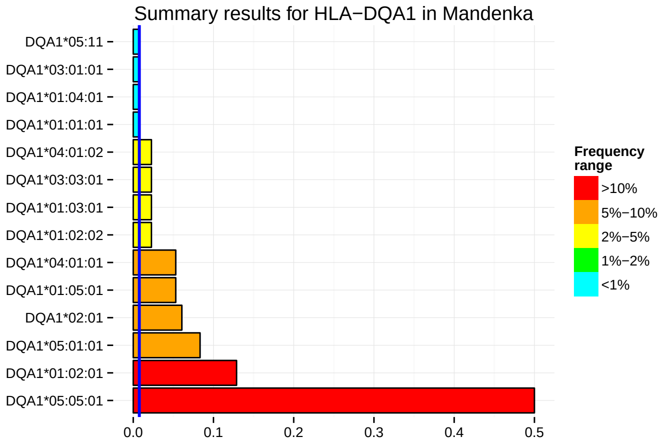

Sample size: 66
Estimated heterozygosity: 0.7149
Hardy−Weinberg equilibrium test p−value: 1
Ewens−Watterson−Slatkin neutrality test: FDR p.adj min: 0.783 max: 0.935

Follows the complete list of alleles, including those with lower
and null frequencies not shown before.

PCR−SSO

NGS−454

	Allele or Haplotype Name	Frequency Estimate
1	11	0.0076
2	9	0.0227
3	8	0.0303
4	5	0.0455
5	2	0.0606
6	7	0.0606
7	6	0.0682
8	4	0.0985
9	3	0.6061

NGS−MiSeq

	Allele or Haplotype Name	Frequency Estimate
1	DQA1*05:11	0.0076
2	DQA1*03:01:01	0.0076
3	DQA1*01:01:01	0.0076
4	DQA1*01:04:01	0.0076
5	DQA1*03:03:01	0.0227
6	DQA1*01:02:02	0.0227
7	DQA1*01:03:01	0.0227
8	DQA1*04:01:02	0.0227
9	DQA1*01:05:01	0.0530
10	DQA1*04:01:01	0.0530
11	DQA1*02:01	0.0606
12	DQA1*05:01:01	0.0833
13	DQA1*01:02:01	0.1288
14	DQA1*05:05:01	0.5000

PCR−SSO

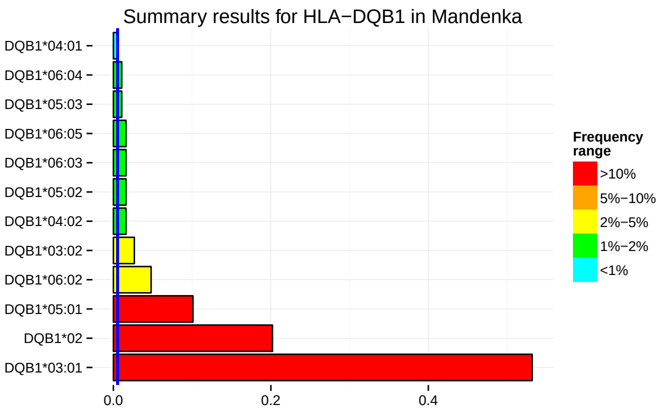

Sample size: 94
Estimated heterozygosity: 0.6617
Hardy−Weinberg equilibrium test p−value: 0.7363483
Ewens−Watterson−Slatkin neutrality test: FDR p.adj min: 0.897 max: 0.969

Follows the complete list of alleles, including those with lower
and null frequencies not shown before.

NGS−454

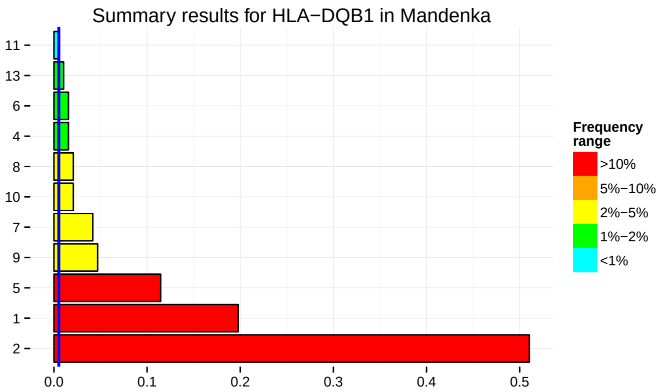

Sample size: 96
Estimated heterozygosity: 0.6818
Hardy−Weinberg equilibrium test p−value: 1
Ewens−Watterson−Slatkin neutrality test: FDR p.adj min: 0.712 max: 0.900

Follows the complete list of alleles, including those with lower
and null frequencies not shown before.

NGS−MiSeq

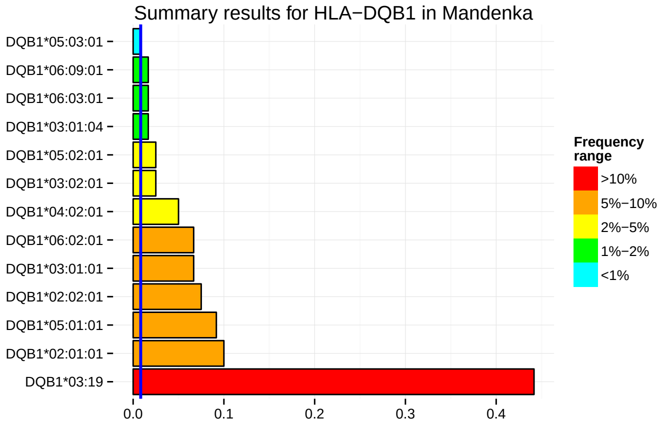

Sample size: 60
Estimated heterozygosity: 0.7673
Hardy−Weinberg equilibrium test p−value: 1
Ewens−Watterson−Slatkin neutrality test: FDR p.adj min: 0.425 max: 0.870

Follows the complete list of alleles, including those with lower
and null frequencies not shown before.

PCR−SSO

	Allele or Haplotype Name	Frequency Estimate
1	DQB1*04:01	0.0053
2	DQB1*05:03	0.0106
3	DQB1*06:04	0.0106
4	DQB1*04:02	0.0160
5	DQB1*06:03	0.0160
6	DQB1*05:02	0.0160
7	DQB1*06:05	0.0160
8	DQB1*03:02	0.0266
9	DQB1*06:02	0.0479
10	DQB1*05:01	0.1011
11	DQB1*02	0.2021
12	DQB1*03:01	0.5319

NGS−454

	Allele or Haplotype Name	Frequency Estimate
1	11	0.0052
2	13	0.0104
3	4	0.0156
4	6	0.0156
5	8	0.0208
6	10	0.0208
7	7	0.0417
8	9	0.0469
9	5	0.1146
10	1	0.1979
11	2	0.5104

NGS−MiSeq

	Allele or Haplotype Name	Frequency Estimate
1	DQB1*05:03:01	0.0083
2	DQB1*03:01:04	0.0167
3	DQB1*06:09:01	0.0167
4	DQB1*06:03:01	0.0167
5	DQB1*03:02:01	0.0250
6	DQB1*05:02:01	0.0250
7	DQB1*04:02:01	0.0500
8	DQB1*03:01:01	0.0667
9	DQB1*06:02:01	0.0667
10	DQB1*02:02:01	0.0750
11	DQB1*05:01:01	0.0917
12	DQB1*02:01:01	0.1000
13	DQB1*03:19	0.4417

PCR−SSO

NGS−454

NGS−MiSeq

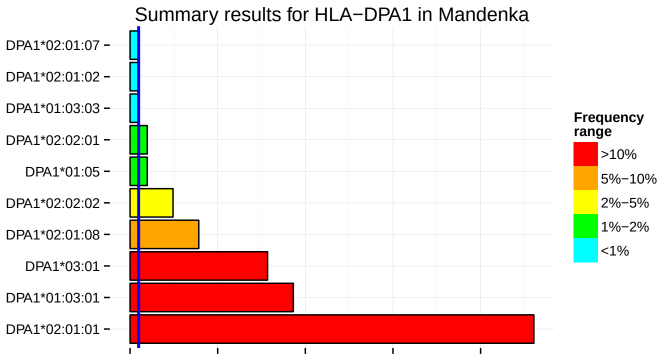

Sample size: 51

Estimated heterozygosity: 0.7187

Hardy−Weinberg equilibrium test p−value: 0.005594244

Ewens−Watterson−Slatkin neutrality test: FDR p.adj min: 0.751 max: 0.920

Follows the complete list of alleles, including those with lower and null frequencies not shown before.

PCR−SSO

NGS−454

NGS−MiSeq

	Allele or Haplotype Name	Frequency Estimate
1	DPA1*01:03:03	0.0098
2	DPA1*02:01:07	0.0098
3	DPA1*02:01:02	0.0098
4	DPA1*01:05	0.0196
5	DPA1*02:02:01	0.0196
6	DPA1*02:02:02	0.0490
7	DPA1*02:01:08	0.0784
8	DPA1*03:01	0.1569
9	DPA1*01:03:01	0.1863
10	DPA1*02:01:01	0.4608

PCR−SSO

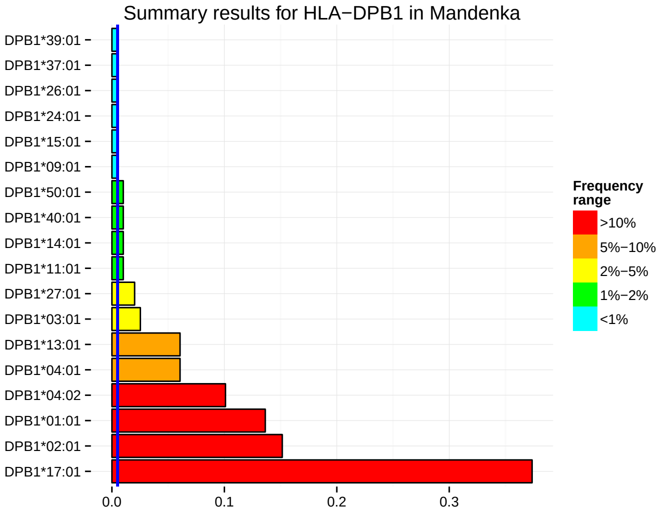

Sample size: 99
Estimated heterozygosity: 0.7996
Hardy−Weinberg equilibrium test p−value: 0.6708691
Ewens−Watterson−Slatkin neutrality test: FDR p.adj min: 0.863 max: 0.975

Follows the complete list of alleles, including those with lower and null frequencies not shown before.

NGS−454

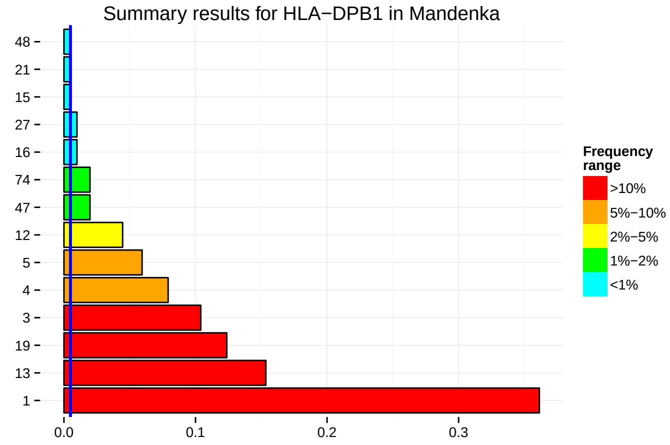

Sample size: 101
Estimated heterozygosity: 0.8068
Hardy−Weinberg equilibrium test p−value: 1
Ewens−Watterson−Slatkin neutrality test: FDR p.adj min: 0.383 max: 0.686

Follows the complete list of alleles, including those with lower and null frequencies not shown before.

NGS−MiSeq

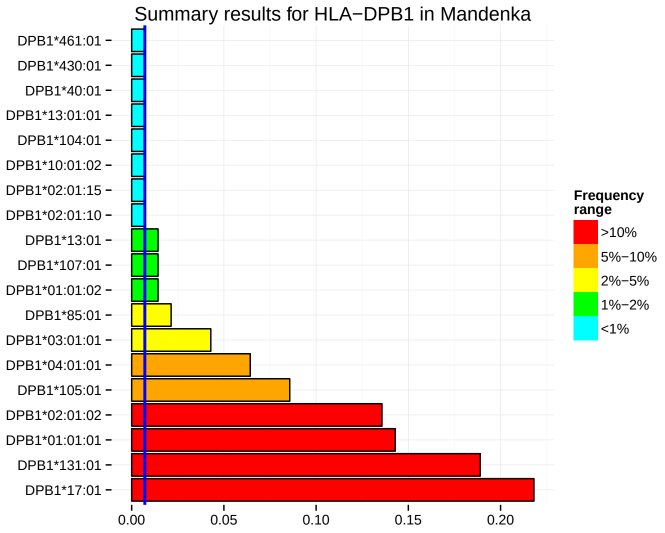

Sample size: 70
Estimated heterozygosity: 0.8631
Hardy−Weinberg equilibrium test p−value: 0.0551156
Ewens−Watterson−Slatkin neutrality test: FDR p.adj min: 0.719 max: 0.897

Follows the complete list of alleles, including those with lower and null frequencies not shown before.

PCR−SSO

	Allele or Haplotype Name	Frequency Estimate
1	DPB1*15:01	0.0051
2	DPB1*09:01	0.0051
3	DPB1*24:01	0.0051
4	DPB1*37:01	0.0051
5	DPB1*39:01	0.0051
6	DPB1*26:01	0.0051
7	DPB1*11:01	0.0101
8	DPB1*14:01	0.0101
9	DPB1*40:01	0.0101
10	DPB1*50:01	0.0101
11	DPB1*27:01	0.0202
12	DPB1*03:01	0.0253
13	DPB1*04:01	0.0606
14	DPB1*13:01	0.0606
15	DPB1*04:02	0.1010
16	DPB1*01:01	0.1364
17	DPB1*02:01	0.1515
18	DPB1*17:01	0.3737

NGS−454

	Allele or Haplotype Name	Frequency Estimate
1	15	0.0050
2	21	0.0050
3	48	0.0050
4	16	0.0099
5	27	0.0099
6	47	0.0198
7	74	0.0198
8	12	0.0446
9	5	0.0594
10	4	0.0792
11	3	0.1040
12	19	0.1238
13	13	0.1535
14	1	0.3614

NGS−MiSeq

	Allele or Haplotype Name	Frequency Estimate
1	DPB1*02:01:15	0.0071
2	DPB1*104:01	0.0071
3	DPB1*13:01:01	0.0071
4	DPB1*02:01:10	0.0071
5	DPB1*10:01:02	0.0071
6	DPB1*40:01	0.0071
7	DPB1*430:01	0.0071
8	DPB1*461:01	0.0071
9	DPB1*01:01:02	0.0143
10	DPB1*107:01	0.0143
11	DPB1*13:01	0.0143
12	DPB1*85:01	0.0214
13	DPB1*03:01:01	0.0429
14	DPB1*04:01:01	0.0643
15	DPB1*105:01	0.0857
16	DPB1*02:01:02	0.1357
17	DPB1*01:01:01	0.1429
18	DPB1*131:01	0.1890
19	DPB1*17:01	0.2181
