# Supplementary material for: Deciphering the fine nucleotide diversity of full HLA class I and class II genes in a well‐documented population from sub‐Saharan Africa
Source: HLA. 2017 Dec 25;91(1):36–51. doi: 10.1111/tan.13180 (PMC5767763; doi:10.1111/tan.13180)
Supplement: Supplementary file 3 — Supplementary Information S03 NGS‐454 assigned sequence numbers with corresponding alleles [file TAN-91-36-s002.pdf]

Supplementary Information S03

NGS-454 Assigned sequence numbers with corresponding alleles

For each locus, each line corresponds to a unique sequence, the number of which is given by "Sequence Number" .

Not all sequences identified are represented here because some were found to be artefacts during the analysis and thus removed from the dataset, e.g. sequence numbers 1,3,7,10 for HLA-DRB1 are missing.

"Number of alleles" is the number of ambiguities reported for this sequence, and "Corresponding alleles" is the list of all possible allele ambiguities (according to IPD-IMGT/HLA database ver 3.25.0).

Locus DRB1

Sequence Number	Number of alleles	Corresponding alleles							
**2**	5	DRB1*07:56	DRB1*07:01:01	DRB1*07:01:18	DRB1*07:33	DRB1*07:34			
**4**	8	DRB1*03:83	DRB1*03:01:01	DRB1*03:01:08	DRB1*03:01:11	DRB1*03:100:02	DRB1*03:104	DRB1*03:50	DRB1*03:68N
**5**	4	DRB3*02:29N	DRB1*14:141	DRB3*02:02:01	DRB3*02:28				
**6**	1	DRB1*13:03:01							
**8**	1	DRB1*01:02:01							
**9**	4	DRB1*04:52	DRB1*04:03:01	DRB1*04:03:03	DRB1*04:181				
**13**	4	DRB1*13:128	DRB1*13:02:01	DRB1*13:02:08	DRB1*13:109				
**17**	1	DRB1*11:02:01							
**19**	8	DRB1*13:190	DRB1*13:01:01	DRB1*13:01:08	DRB1*13:105	DRB1*13:112	DRB1*13:117	DRB1*13:166	DRB1*13:186
**20**	3	DRB1*08:59	DRB1*08:04:01	DRB1*08:04:04					
**21**	1	DRB1*13:04							
**23**	2	DRB1*10:01:04	DRB1*10:01:01						
**26**	3	DRB1*04:05:04	DRB1*04:05:01	DRB1*04:05:03					
**27**	7	DRB1*11:97	DRB1*11:01:01	DRB1*11:01:02	DRB1*11:01:06	DRB1*11:01:08	DRB1*11:01:21	DRB1*11:100	
**34**	1	DRB1*08:06							
**35**	3	DRB1*16:35	DRB1*16:02:01	DRB1*16:22					
**37**	4	DRB1*12:17	DRB1*12:01:01	DRB1*12:06	DRB1*12:10				
**38**	3	DRB1*09:21	DRB1*09:01:02	DRB1*09:09					
**39**	8	DRB1*14:54:01	DRB1*14:01:01	DRB1*14:01:04	DRB1*14:113	DRB1*14:114	DRB1*14:125	DRB1*14:142	DRB1*14:157
**49**	8	DRB1*01:71	DRB1*01:01:01	DRB1*01:01:05	DRB1*01:01:25	DRB1*01:01:29	DRB1*01:50	DRB1*01:63	DRB1*01:67
**53**	1	DRB1*11:02:01							
**54**	8	DRB1*13:190	DRB1*13:01:01	DRB1*13:01:08	DRB1*13:105	DRB1*13:112	DRB1*13:117	DRB1*13:166	DRB1*13:186
**57**	1	DRB1*03:02:01							
**58**	7	DRB1*11:97	DRB1*11:01:01	DRB1*11:01:02	DRB1*11:01:06	DRB1*11:01:08	DRB1*11:01:21	DRB1*11:100	
**59**	1	DRB1*03:02:01							
**60**	1	DRB1*08:06							

Locus DQA1

Sequence Number	Number of alleles	Corresponding alleles							
**3**	14	DQA1*05:11	DQA1*05:01:01:01	DQA1*05:01:01:02	DQA1*05:01:01:03	DQA1*05:03	DQA1*05:05:01:01	DQA1*05:05:01:02	DQA1*05:05:01:03
		DQA1*05:05:01:04	DQA1*05:05:01:05	DQA1*05:06	DQA1*05:07	DQA1*05:08	DQA1*05:09		
**4**	8	DQA1*01:11	DQA1*01:02:01:01	DQA1*01:02:01:02	DQA1*01:02:01:03	DQA1*01:02:01:04	DQA1*01:02:02	DQA1*01:02:03	DQA1*01:02:04
**5**	8	DQA1*01:11	DQA1*01:02:01:01	DQA1*01:02:01:02	DQA1*01:02:01:03	DQA1*01:02:01:04	DQA1*01:02:02	DQA1*01:02:03	DQA1*01:02:04
**6**	4	DQA1*04:02	DQA1*04:01:01	DQA1*04:01:02:01	DQA1*04:01:02:02				
**7**	12	DQA1*01:07Q	DQA1*01:01:01:01	DQA1*01:01:01:02	DQA1*01:01:01:03	DQA1*01:01:02	DQA1*01:04:01:01	DQA1*01:04:01:02	DQA1*01:04:01:03
		DQA1*01:04:01:04	DQA1*01:04:02	DQA1*01:05:01	DQA1*01:05:02				
**8**	6	DQA1*03:03:02	DQA1*03:01:01	DQA1*03:02	DQA1*03:03:01:01	DQA1*03:03:01:02	DQA1*03:03:01:03		
**9**	7	DQA1*01:10	DQA1*01:03:01:01	DQA1*01:03:01:02	DQA1*01:03:01:03	DQA1*01:03:01:04	DQA1*01:03:01:05	DQA1*01:03:01:06	
**11**	12	DQA1*01:07Q	DQA1*01:01:01:01	DQA1*01:01:01:02	DQA1*01:01:01:03	DQA1*01:01:02	DQA1*01:04:01:01	DQA1*01:04:01:02	DQA1*01:04:01:03
		DQA1*01:04:01:04	DQA1*01:04:02	DQA1*01:05:01	DQA1*01:05:02				

Locus DQB1

Sequence Number	Number of alleles	Corresponding alleles							
**1**	16	DQB1*02:02:01	DQB1*02:01:01	DQB1*02:01:08	DQB1*02:01:10	DQB1*02:02:02	DQB1*02:04	DQB1*02:06	DQB1*02:09
		DQB1*02:10	DQB1*02:12	DQB1*02:20N	DQB1*02:29	DQB1*02:34	DQB1*02:48	DQB1*02:59	DQB1*02:61
**2**	35	DQB1*03:01:01	DQB1*03:01:04	DQB1*03:01:05	DQB1*03:01:09	DQB1*03:01:10	DQB1*03:01:11	DQB1*03:01:12	DQB1*03:01:20
		DQB1*03:01:26	DQB1*03:09	DQB1*03:115	DQB1*03:116	DQB1*03:120	DQB1*03:127	DQB1*03:150	DQB1*03:157
		DQB1*03:158	DQB1*03:164	DQB1*03:165	DQB1*03:169	DQB1*03:182	DQB1*03:19	DQB1*03:191	DQB1*03:21
		DQB1*03:22	DQB1*03:24	DQB1*03:29	DQB1*03:35	DQB1*03:42	DQB1*03:49	DQB1*03:50	DQB1*03:51
		DQB1*03:52	DQB1*03:84N	DQB1*03:94					
**4**	8	DQB1*03:02:01	DQB1*03:02:09	DQB1*03:02:12	DQB1*03:02:17	DQB1*03:106	DQB1*03:190	DQB1*03:32	DQB1*03:85
**5**	9	DQB1*05:01:01	DQB1*05:18	DQB1*05:27	DQB1*05:31	DQB1*05:32	DQB1*05:45	DQB1*05:62	DQB1*05:74
		DQB1*05:84							
**6**	17	DQB1*05:102	DQB1*05:02:01	DQB1*05:02:03	DQB1*05:02:07	DQB1*05:02:11	DQB1*05:14	DQB1*05:17	DQB1*05:35
		DQB1*05:36	DQB1*05:37	DQB1*05:46	DQB1*05:47	DQB1*05:57	DQB1*05:79	DQB1*05:83	DQB1*05:87Q
		DQB1*05:90N							
**7**	18	DQB1*06:02:01	DQB1*06:02:04	DQB1*06:02:12	DQB1*06:02:23	DQB1*06:109	DQB1*06:11:01	DQB1*06:111	DQB1*06:112N
		DQB1*06:115	DQB1*06:116	DQB1*06:117	DQB1*06:127	DQB1*06:131	DQB1*06:175	DQB1*06:176	DQB1*06:47
		DQB1*06:72	DQB1*06:84						
**8**	3	DQB1*06:09:01	DQB1*06:22:02	DQB1*06:88					
**9**	4	DQB1*04:23	DQB1*04:02:01	DQB1*04:04	DQB1*04:13				
**10**	5	DQB1*06:03:01	DQB1*06:110	DQB1*06:14:01	DQB1*06:41	DQB1*06:44			
**11**	8	DQB1*06:86	DQB1*06:04:01	DQB1*06:34	DQB1*06:36	DQB1*06:38	DQB1*06:39	DQB1*06:52	DQB1*06:69
**13**	12	DQB1*05:03:01	DQB1*05:03:03	DQB1*05:03:04	DQB1*05:03:09	DQB1*05:08	DQB1*05:10	DQB1*05:38	DQB1*05:41N
		DQB1*05:42	DQB1*05:56	DQB1*05:78	DQB1*05:96				

Locus DPB1

Sequence Number	Number of alleles	Corresponding alleles						
**1**	4	DPB1*460:01	DPB1*168:01	DPB1*17:01	DPB1*131:01			
**3**	3	DPB1*463:01	DPB1*105:01	DPB1*04:02:01				
**4**	7	DPB1*459:01	DPB1*415:01	DPB1*350:01	DPB1*126:01	DPB1*464:01	DPB1*04:01:31	DPB1*04:01:01
**5**	3	DPB1*133:01	DPB1*13:01:01	DPB1*107:01				
**12**	5	DPB1*351:01	DPB1*124:01	DPB1*03:01:08	DPB1*104:01	DPB1*03:01:01		
**13**	6	DPB1*461:01	DPB1*416:01	DPB1*414:01	DPB1*352:01	DPB1*141:01	DPB1*02:01:02	
**15**	1	DPB1*15:01:01						
**16**	1	DPB1*40:01						
**19**	3	DPB1*462:01	DPB1*417:01	DPB1*01:01:01				
**21**	1	DPB1*10:01						
**27**	1	DPB1*11:01:01						
**47**	2	DPB1*162:01	DPB1*01:01:02					
**48**	1	DPB1*39:01						
**74**	1	DPB1*85:01						
