# Supplementary material for: Deciphering the fine nucleotide diversity of full HLA class I and class II genes in a well‐documented population from sub‐Saharan Africa
Source: HLA. 2017 Dec 25;91(1):36–51. doi: 10.1111/tan.13180 (PMC5767763; doi:10.1111/tan.13180)
Supplement: Supplementary file 4 — Supplementary Information S04 Global linkage disequilibrium between the 8 HLA loci [file TAN-91-36-s007.pdf]

# Supplementary Information S04

**Table S04-1: Global linkage disequilibrium between the 8 HLA loci.**

Both parametric and non-parametric tests have been used with the GENE[RATE] computer tools.Significant values are in bold ($p < 0.05$).

| P-values for parametric test | | | | | | | | |
|---|---|---|---|---|---|---|---|---|
| | **HLA-A** | **HLA-B** | **HLA-C** | **HLA-DRB1** | **HLA-DQA1** | **HLA-DQB1** | **HLA-DPA1** | **HLA-DPB1** |
| **HLA-A** | — | 1 | 1 | 1 | 1 | 0.99 | 0.99 | 1 |
| **HLA-B** | 1 | — | 1 | 1 | 1 | 1 | 1 | 1 |
| **HLA-C** | 1 | 1 | — | 0.99 | 0.99 | 0.99 | 0.99 | 1 |
| **HLA-DRB1** | 1 | 1 | 0.37 | — | **0.002** | 0.07 | 0.99 | 1 |
| **HLA-DQA1** | 1 | 1 | 0.6 | **<0.01** | — | **<0.01** | 0.99 | 1 |
| **HLA-DQB1** | 0.48 | 1 | 0.27 | **<0.01** | **<0.01** | — | 0.99 | 1 |
| **HLA-DPA1** | 0.79 | 1 | 0.56 | 0.05 | 0.12 | 0.17 | — | **0.00081** |
| **HLA-DPB1** | 1 | 1 | 1 | 1 | 1 | 1 | **<0.01** | — |
| | **HLA-A** | **HLA-B** | **HLA-C** | **HLA-DRB1** | **HLA-DQA1** | **HLA-DQB1** | **HLA-DPA1** | **HLA-DPB1** |
| **P-values for non-parametric test** | | | | | | | | |

Both parametric and non-parametric tests have been used with the GENE[RATE] computer tools.Significant values are in bold ($p < 0.05$).

**Table S04-2: Haplotype frequencies and linkage disequilibrium**

Only haplotypes in positive significant linkage disequilibrium (i.e. standardized residuals > 1.96, and observed frequency > 5%) are listed, in decreasing order of the standardized residuals. D is the value of the linkage disequilibrium coefficient, corresponding of the difference between the observed and expected frequencies. Test were performed with the GENE[RATE] computer tools.

| Haplotypes | Observed frequencies | Expected frequencies | Linkage disquilibrium coefficient D | Standardized Residuals |
|---|---|---|---|---|
| DQA1*04:01:01~DQB1*04:02:01 | 0,053 | 0,003 | 0,050 | 10,101 |
| DQA1*02:01~DRB1*07:01:01 | 0,057 | 0,004 | 0,053 | 9,732 |
| DQB1*06:02:01~DRB1*08:06 | 0,060 | 0,004 | 0,056 | 9,354 |
| DQA1*05:01:01~DQB1*02:01:01 | 0,088 | 0,008 | 0,079 | 9,157 |
| DQA1*05:01:01~DRB1*03:01:01 | 0,081 | 0,008 | 0,073 | 9,095 |
| DQB1*02:01:01~DRB1*03:01:01 | 0,078 | 0,009 | 0,069 | 7,801 |
| DQA1*01:05:01~DQB1*05:01:01 | 0,053 | 0,005 | 0,048 | 7,104 |
| DQA1*01:02:01~DQB1*06:02:01 | 0,070 | 0,009 | 0,062 | 7,051 |
| DPA1*03:01~DPB1*105:01 | 0,108 | 0,017 | 0,091 | 6,998 |
| DQA1*01:02:01~DRB1*08:06 | 0,057 | 0,007 | 0,050 | 6,666 |
| DPA1*02:01:08~DPB1*01:01:01 | 0,078 | 0,011 | 0,068 | 6,548 |
| DQB1*02:02:01~DRB1*07:01:01 | 0,052 | 0,006 | 0,046 | 6,307 |
| DPA1*03:01~DRB1*08:06 | 0,073 | 0,012 | 0,061 | 5,366 |
| DPA1*01:03:01~DPB1*02:01:02 | 0,108 | 0,026 | 0,082 | 5,125 |
| A*30:02:01~DQA1*01:02:01 | 0,068 | 0,015 | 0,053 | 4,873 |
| DPB1*131:01~DRB1*13:04 | 0,152 | 0,056 | 0,096 | 4,500 |
| A*30:02:01~DPA1*03:01 | 0,069 | 0,015 | 0,053 | 4,301 |
| DQB1*03:19~DRB1*13:04 | 0,259 | 0,119 | 0,140 | 4,092 |
| DQA1*05:05:01~DQB1*03:19 | 0,430 | 0,228 | 0,202 | 3,963 |
| DPA1*03:01~DQB1*06:02:01 | 0,065 | 0,015 | 0,050 | 3,875 |
| DQA1*05:05:01~DRB1*13:04 | 0,282 | 0,148 | 0,135 | 3,609 |
| DPA1*01:03:01~DRB1*03:01:01 | 0,052 | 0,012 | 0,039 | 3,426 |
| DPA1*02:01:01~DPB1*131:01 | 0,201 | 0,097 | 0,104 | 3,198 |
| DPA1*02:01:01~DPB1*17:01 | 0,201 | 0,097 | 0,104 | 3,198 |
| C*16:01:01~DPB1*01:01:01 | 0,065 | 0,020 | 0,044 | 3,198 |
| DPB1*131:01~DQA1*05:05:01 | 0,171 | 0,097 | 0,074 | 2,616 |
| A*30:02:01~C*04:01:01 | 0,055 | 0,020 | 0,035 | 2,565 |
| A*33:03:01~DRB1*13:04 | 0,069 | 0,031 | 0,039 | 2,454 |
| A*33:03:01~DPB1*17:01 | 0,057 | 0,024 | 0,032 | 2,428 |
| DPA1*02:01:01~DRB1*13:04 | 0,206 | 0,117 | 0,090 | 2,418 |
| A*23:01:01~B*35:01:01 | 0,065 | 0,029 | 0,036 | 2,409 |
| DPA1*03:01~DQA1*01:02:01 | 0,061 | 0,025 | 0,036 | 2,238 |
| B*35:01:01~C*16:01:01 | 0,065 | 0,028 | 0,037 | 2,237 |
| DQA1*05:05:01~DRB1*11:01:02 | 0,089 | 0,045 | 0,044 | 2,236 |
| C*04:01:01~DPB1*105:01 | 0,052 | 0,021 | 0,031 | 2,217 |
| B*35:01:01~DPB1*131:01 | 0,067 | 0,032 | 0,035 | 2,195 |
| DPB1*131:01~DQB1*03:19 | 0,148 | 0,086 | 0,061 | 2,177 |
| DQB1*03:19~DRB1*11:02:01 | 0,069 | 0,033 | 0,036 | 2,059 |
| B*52:01:02~DQB1*03:19 | 0,052 | 0,023 | 0,029 | 2,046 |
