# Supplementary material for: Deciphering the fine nucleotide diversity of full HLA class I and class II genes in a well‐documented population from sub‐Saharan Africa
Source: HLA. 2017 Dec 25;91(1):36–51. doi: 10.1111/tan.13180 (PMC5767763; doi:10.1111/tan.13180)
Supplement: Supplementary file 5 — Supplementary Information S05 Statistics computed for the assessment of selective neutrality in each gene region [file TAN-91-36-s006.pdf]

Supplementary Information S05

Statistics computed for the assessment of selective neutrality in each gene region

Locus	Gene region	π ± sd	S	Tajima's D	pVal	adj.pVal	dN	dS	dN/dS	Z	N seq / N tot
HLA-A	Exon1	0.022 ± 0.014	8	0,30	0,66	0,75	0,0201	0,0284	0,7075	-0,33	174 / 174
HLA-B	Exon1	0.047 ± 0.027	12	1,54	0,94	0,24	0,0483	0,0541	0,8933	-0,16	166 / 166
HLA-C	Exon1	0.023 ± 0.015	6	1,21	0,88	0,41	0,0249	0,0000	*nan*	*nan*	163 / 166
HLA-DPA1	Exon1	0	0	*nan*	*nan*	*nan*	*nan*	*nan*	*nan*	*nan*	102 / 102
HLA-DQA1	Exon1	0.027 ± 0.017	7	1,70	0,96	0,20	0,0205	0,0507	0,4044	-0,86	158 / 164
HLA-DQB1	Exon1	0.036 ± 0.02	14	1,37	0,93	0,28	0,0337	0,0491	0,6871	-0,47	138 / 154
HLA-DRB1	Exon1	0.014 ± 0.01	8	-0,34	0,43	0,86	0,0151	0,0128	1,1850	0,39	82 / 162
HLA-A	Exon2	0.046 ± 0.023	81	-0,42	0,37	0,80	0,0505	0,0428	1,1786	0,43	172 / 174
HLA-B	Exon2	0.047 ± 0.023	42	2,02	**0,99**	0,09	0,0495	0,0458	1,0804	0,18	164 / 166
HLA-C	Exon2	0.025 ± 0.013	21	2,26	**0,98**	0,11	0,0248	0,0291	0,8529	-0,29	164 / 166
HLA-DPA1	Exon2	0.02 ± 0.011	13	2,52	**0,99**	0,07	0,0151	0,0406	0,3724	-1,16	102 / 102
HLA-DPB1	Exon2	0.031 ± 0.016	21	3,24	**1,00**	**0,04**	0,0386	0,0103	3,7641	**2,56**	166 / 166
HLA-DQA1	Exon2	0.062 ± 0.031	46	2,16	**0,98**	0,13	0,0565	0,0808	0,6992	-0,88	156 / 164
HLA-DQB1	Exon2	0.064 ± 0.032	54	2,38	**0,99**	0,08	0,0720	0,0602	1,1956	0,55	152 / 154
HLA-DRB1	Exon2	0.062 ± 0.031	62	1,48	0,95	0,23	0,0689	0,0567	1,2162	0,57	141 / 162
**HLA-A**	**Exon2 – ARS**	0.083 ± 0.044	26	0,57	0,77	0,61	0,1050	0,0438	2,3940	1,19	172 / 174
**HLA-B**	**Exon2 – ARS**	0.111 ± 0.058	22	2,44	**1,00**	0,06	0,1603	0,0212	7,5661	**3,15**	164 / 166
**HLA-C**	**Exon2 – ARS**	0.052 ± 0.03	9	2,72	**1,00**	**0,04**	0,0642	0,0267	2,4078	1,16	164 / 166
**HLA-DPA1**	**Exon2 – ARS**	0.032 ± 0.02	6	1,49	0,91	0,33	0,0420	0,0017	25,3267	1,49	102 / 102
**HLA-DPB1**	**Exon2 – ARS**	0.05 ± 0.028	8	3,73	**1,00**	**<0.01**	0,0674	0,0000	*nan*	*nan*	166 / 166
**HLA-DQA1**	**Exon2 – ARS**	0.072 ± 0.04	14	1,92	0,97	0,16	0,0797	0,0799	0,9977	0,00	156 / 164
**HLA-DQB1**	**Exon2 – ARS**	0.133 ± 0.068	31	2,33	**0,99**	0,06	0,1873	0,0742	2,5251	1,89	152 / 154
**HLA-DRB1**	**Exon2 – ARS**	0.137 ± 0.07	35	1,87	0,97	0,14	0,1841	0,0875	2,1049	1,53	144 / 162
**HLA-A**	**Exon2 – non ARS**	0.034 ± 0.018	55	-0,89	0,21	0,58	0,0333	0,0432	0,7701	-0,55	172 / 174
**HLA-B**	**Exon2 – non ARS**	0.026 ± 0.014	20	1,24	0,91	0,33	0,0175	0,0545	0,3216	-1,57	164 / 166
**HLA-C**	**Exon2 – non ARS**	0.016 ± 0.009	12	1,39	0,94	0,26	0,0124	0,0301	0,4124	-1,04	164 / 166
**HLA-DPA1**	**Exon2 – non ARS**	0.016 ± 0.009	7	2,73	**1,00**	0,06	0,0066	0,0537	0,1233	-1,64	102 / 102
**HLA-DPB1**	**Exon2 – non ARS**	0.023 ± 0.013	13	2,25	**0,98**	0,11	0,0271	0,0138	1,9596	1,29	166 / 166
**HLA-DQA1**	**Exon2 – non ARS**	0.058 ± 0.03	32	2,05	**0,99**	0,10	0,0490	0,0820	0,5981	-1,09	156 / 164
**HLA-DQB1**	**Exon2 – non ARS**	0.037 ± 0.019	23	2,11	**0,98**	0,13	0,0337	0,0558	0,6033	-0,99	154 / 154
**HLA-DRB1**	**Exon2 – non ARS**	0.032 ± 0.017	27	0,76	0,81	0,54	0,0295	0,0461	0,6393	-0,82	141 / 162
**HLA-A**	**Exon3**	0.039 ± 0.02	34	2,29	**0,99**	0,10	0,0442	0,0280	1,5795	1,21	172 / 174
**HLA-B**	**Exon3**	0.039 ± 0.02	37	1,82	0,96	0,20	0,0400	0,0401	0,9978	-0,01	164 / 166
**HLA-C**	**Exon3**	0.03 ± 0.015	28	1,85	0,96	0,18	0,0301	0,0316	0,9530	-0,10	164 / 166
**HLA-A**	**Exon3 – ARS**	0.114 ± 0.06	17	2,85	**1,00**	**0,03**	0,1548	0,0421	3,6759	**3,05**	172 / 174
**HLA-B**	**Exon3 – ARS**	0.124 ± 0.065	20	2,44	**0,99**	0,09	0,1622	0,0717	2,2616	1,32	164 / 166
**HLA-C**	**Exon3 – ARS**	0.094 ± 0.051	13	3,05	**1,00**	**0,02**	0,1254	0,0281	4,4602	**2,25**	164 / 166
**HLA-A**	**Exon3 – non ARS**	0.02 ± 0.011	17	1,33	0,93	0,29	0,0194	0,0250	0,7764	-0,40	172 / 174
**HLA-B**	**Exon3 – non ARS**	0.018 ± 0.01	17	0,78	0,83	0,52	0,0129	0,0337	0,3839	-1,42	164 / 166
**HLA-C**	**Exon3 – non ARS**	0.014 ± 0.008	15	0,42	0,74	0,66	0,0080	0,0329	0,2421	-1,76	164 / 166
HLA-DPA1	Exon3	0.011 ± 0.006	11	1,15	0,89	0,38	0,0067	0,0260	0,2573	-1,52	102 / 102
HLA-DPB1	Exon3	0.013 ± 0.007	7	4,02	**1,00**	**<0.01**	0,0047	0,0405	0,1164	-1,84	166 / 166
HLA-DQA1	Exon3	0.016 ± 0.009	14	2,01	**0,98**	0,11	0,0142	0,0227	0,6246	-0,71	156 / 164
HLA-DQB1	Exon3	0.02 ± 0.01	18	1,90	**0,98**	0,13	0,0145	0,0383	0,3787	-1,53	154 / 154
HLA-DRB1	Exon3	0.013 ± 0.007	25	-0,54	0,33	0,74	0,0089	0,0275	0,3224	-1,93	153 / 162
HLA-A	Exon4	0.029 ± 0.015	23	2,73	**0,99**	0,06	0,0167	0,0758	0,2202	**-2,62**	172 / 174
HLA-B	Exon4	0.006 ± 0.004	7	0,81	0,81	0,54	0,0028	0,0172	0,1611	-1,31	166 / 166
HLA-C	Exon4	0.018 ± 0.01	20	1,07	0,88	0,42	0,0133	0,0346	0,3843	-1,61	166 / 166
HLA-DPA1	Exon4	0.006 ± 0.005	3	1,05	0,84	0,50	0,0046	0,0101	0,4566	-0,49	102 / 102
HLA-DPB1	Exon4	0.005 ± 0.004	4	-0,48	0,39	0,80	0,0064	0,0000	*nan*	*nan*	166 / 166
HLA-DQA1	Exon4	0.016 ± 0.01	13	0,14	0,61	0,80	0,0086	0,0372	0,2308	-1,69	156 / 164
HLA-DQB1	Exon4	0.029 ± 0.017	9	2,24	**0,98**	0,13	0,0167	0,0750	0,2220	-1,52	153 / 154
HLA-DRB1	Exon4	0.002 ± 0.003	2	-0,57	0,33	0,73	0,0000	0,0079	0,0000	-1,30	150 / 162
HLA-A	Exon5	0.037 ± 0.02	14	1,79	0,97	0,15	0,0378	0,0382	0,9879	-0,02	172 / 174
HLA-B	Exon5	0.012 ± 0.008	6	0,66	0,80	0,57	0,0108	0,0163	0,6639	-0,37	166 / 166
HLA-C	Exon5	0.033 ± 0.018	17	0,79	0,82	0,54	0,0302	0,0458	0,6582	-0,74	166 / 166
HLA-DPB1	Exon5	0.007 ± 0.012	1	-0,32	0,31	0,73	0,0000	0,0344	0,0000	-0,78	92 / 166
HLA-DQB1	Exon5	0 ± 0	0	*nan*	*nan*	*nan*	*nan*	*nan*	*nan*	*nan*	154 / 154
HLA-DRB1	Exon5	0.025 ± 0.022	2	0,78	0,82	0,54	0,0413	0,0000	*nan*	*nan*	87 / 162
HLA-A	Exon6	0.025 ± 0.02	3	0,98	0,85	0,48	0,0177	0,0532	0,3325	-0,40	170 / 174
HLA-B	Exon6	0 ± 0	0	*nan*	*nan*	*nan*	*nan*	*nan*	*nan*	*nan*	166 / 166
HLA-C	Exon6	0.003 ± 0.006	1	-0,43	0,30	0,71	0,0047	0,0000	*nan*	*nan*	164 / 166
HLA-DQB1	Exon6	0.026 ± 0.028	1	0,98	0,87	0,42	0,0000	0,1870	0,0000	-1,12	95 / 154
HLA-DRB1	Exon6	0.005 ± 0.011	2	-1,31	0,05	0,20	0,0037	0,0467	0,0789	-1,78	86 / 162
HLA-A	Exon7	0.01 ± 0.01	2	0,55	0,77	0,61	0,0116	0,0093	1,2398	0,14	170 / 174
HLA-B	Exon7	0.008 ± 0.009	1	1,14	0,89	0,38	0,0000	0,0000	*nan*	*nan*	166 / 166
HLA-C	Exon7	0.009 ± 0.009	2	0,30	0,68	0,73	0,0061	0,0000	*nan*	*nan*	161 / 166
HLA-A	Exon8	0 ± 0	0	*nan*	*nan*	*nan*	*nan*	*nan*	*nan*	*nan*	170 / 174
HLA-C	Exon8	0 ± 0	0	*nan*	*nan*	*nan*	*nan*	*nan*	*nan*	*nan*	165 / 166
HLA-A	Intron1	0.042 ± 0.023	14	3,02	***1,00***	**<0.01**	-	-	-	-	138 / 174
HLA-B	Intron1	0.019 ± 0.012	18	-0,64	0,29	0,71	-	-	-	-	157 / 166
HLA-C	Intron1	0.017 ± 0.01	10	0,53	0,75	0,64	-	-	-	-	164 / 166
HLA-DPA1	Intron1	0.002 ± 0.001	23	0,25	0,66	0,75	-	-	-	-	38 / 102
HLA-DQB1	Intron1	0.001 ± 0.001	4	1,65	0,97	0,17	-	-	-	-	23 / 154

HLA-A	Intron2	0.027 ± 0.014	25	1,30	0,92	0,32	-	-	-	-	172 / 174
HLA-B	Intron2	0.01 ± 0.006	13	0,07	0,60	0,80	-	-	-	-	149 / 166
HLA-C	Intron2	0.012 ± 0.007	11	1,16	0,88	0,41	-	-	-	-	101 / 166
HLA-DPA1	Intron2	0.016 ± 0.009	17	1,87	0,97	0,15	-	-	-	-	102 / 102
HLA-DPB1	Intron2	0.001 ± 0	17	-0,93	0,19	0,54	-	-	-	-	30 / 166
HLA-DQA1	Intron2	0.003 ± 0.002	4	0,38	0,69	0,73	-	-	-	-	36 / 164
HLA-DQB1	Intron2	0 ± 0	0	*nan*	*nan*	*nan*	-	-	-	-	7 / 154
HLA-A	Intron3	0.018 ± 0.009	30	2,77	**1,00**	0,05	-	-	-	-	157 / 174
HLA-B	Intron3	0.003 ± 0.002	13	-0,47	0,39	0,80	-	-	-	-	132 / 166
HLA-C	Intron3	0.013 ± 0.007	37	0,48	0,77	0,61	-	-	-	-	164 / 166
HLA-DPA1	Intron3	0.013 ± 0.008	7	2,44	**0,99**	0,08	-	-	-	-	102 / 102
HLA-DPB1	Intron3	0.013 ± 0.007	19	2,98	**1,00**	0,05	-	-	-	-	166 / 166
HLA-DQB1	Intron3	0.022 ± 0.011	33	2,36	**0,99**	0,09	-	-	-	-	103 / 154
HLA-DRB1	Intron3	0.024 ± 0.012	52	0,97	0,87	0,42	-	-	-	-	28 / 162
HLA-A	Intron4	0.002 ± 0.002	1	-0,25	0,32	0,73	-	-	-	-	81 / 174
HLA-B	Intron4	0.011 ± 0.008	4	0,83	0,82	0,54	-	-	-	-	166 / 166
HLA-C	Intron4	0.03 ± 0.017	17	0,55	0,77	0,61	-	-	-	-	157 / 166
HLA-DPB1	Intron4	0.033 ± 0.017	25	3,68	**1,00**	**0,00**	-	-	-	-	98 / 166
HLA-DQB1	Intron4	0.018 ± 0.009	27	2,11	**0,98**	0,11	-	-	-	-	103 / 154
HLA-A	Intron5	0.017 ± 0.009	21	2,31	**0,99**	0,08	-	-	-	-	81 / 174
HLA-B	Intron5	0.008 ± 0.005	18	0,49	0,75	0,64	-	-	-	-	165 / 166
HLA-C	Intron5	0.005 ± 0.003	11	0,14	0,62	0,80	-	-	-	-	136 / 166
HLA-DQB1	Intron5	0.009 ± 0.005	21	0,86	0,83	0,53	-	-	-	-	60 / 154
HLA-A	Intron6	0.025 ± 0.014	18	0,33	0,68	0,73	-	-	-	-	170 / 174
HLA-B	Intron6	0.007 ± 0.006	5	-0,37	0,40	0,80	-	-	-	-	161 / 166
HLA-C	Intron6	0.013 ± 0.009	6	0,65	0,77	0,61	-	-	-	-	166 / 166
HLA-A	Intron7	0.028 ± 0.015	14	2,30	**0,99**	0,07	-	-	-	-	132 / 174
HLA-C	Intron7	0.012 ± 0.008	10	0,34	0,73	0,68	-	-	-	-	149 / 166

Only sequences without gaps are considered here (at the exception of DQA1 exon 2 codon 56), the total number of sequences matching this criterion is reported in the last column along with the total number of sequences (N seq / N tot). Significant values (p<0.05) are in bold. π ± sd: genetic diversity ± 1 standard deviation; S: number of observed segregating sites; pVal: p-value associated to Tajima's D tests for selective neutrality; adj.pVal: adjusted pValue (False Discovery Rate method) associated to Tajima's D tests for selective neutrality (as Tajima's test is a two tailed test, pValue were transformed before correction for multiple tests, see Material & Methods); dN: proportion of non-synonymous substitutions; dS: proportion of synonymous substitutions; dN/dS: ratio of non-synonymous to synonymous substitutions; Z: Z statistics assessing the significance of dN/dS Ratio (significant at α=5% when Z is oustide the [-1.96;1.96] interval); nan: not computable due to 0 values; "-": not computable on introns.
