# Supplementary material for: Deciphering the fine nucleotide diversity of full HLA class I and class II genes in a well‐documented population from sub‐Saharan Africa
Source: HLA. 2017 Dec 25;91(1):36–51. doi: 10.1111/tan.13180 (PMC5767763; doi:10.1111/tan.13180)
Supplement: Supplementary file 6 — Supplementary Information S06 Principal Component Analysis, axes 1 and 3 [file TAN-91-36-s004.pdf]

Supplementary Information S06

Principal Component Analysis, axes 1 and 3

Principal component analysis (axes 1 and 3, explaining respectively 60% and 11% of the total variance) based on Tajima's D, nucleotide diversity π, frequency of segregating sites S.freq, number of non-synonymous dN and synonymous dS nucleotides at exons 2, 3 and 4 of loci HLA-A, -B, - C, -DRB1, -DQA1, -DQB1, -DPA1, -DPB1 and exons 5 of loci HLA-A, -B, -C. Symbols D, D_1, D_2, D_3 represent Tajima's D estimated on the whole gene region and at the 1st, 2nd and 3rd nucleotide of each codon, respectively. Similarly, π , π_1, π_2 and π_3 as well as S.freq, S.freq_1, S.freq_2 and S.freq_3 represent the nucleotide diversity π and the frequency of segregating sites S estimated on the whole gene region and at nucleotide positions 1, 2 and 3 of each codon, respectively. Grey boxes correspond to ARS codons, white boxes to non-ARS codons. The inset graph at the bottom left represents the correlations between the projections of the variables (for each pair of variables, the correlation is measured by the cosine of the angle of the two variable vectors) on the plan of the PCA.

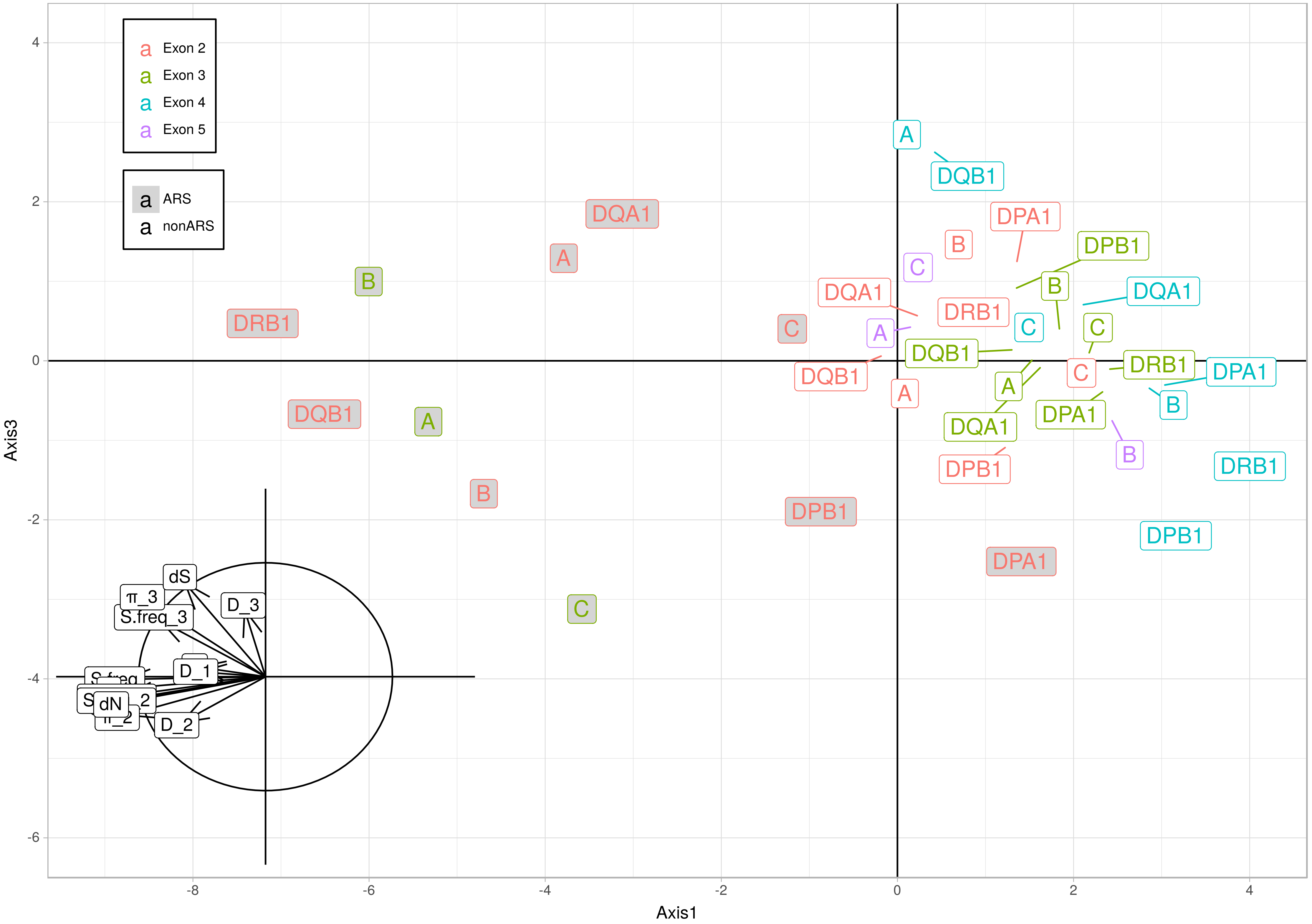

Exon 2
Exon 3
Exon 4
Exon 5
ARS
nonARS
Axis1
Axis3
4
2
0
-2
-4
-6
-8
-6
-4
-2
0
2
4
DQA1
DRB1
DQB1
DPB1
DPA1
DRB1
DQA1
DQB1
DPA1
DPB1
DQB1
DPB1
DQA1
DRB1
DQB1
DPA1
DQA1
DRB1
DPA1
DRB1
DPB1
A
B
C
dS
π_3
D_3
S.freq_3
D_1
dN
D_2
