# Supplementary material for: Deciphering the fine nucleotide diversity of full HLA class I and class II genes in a well‐documented population from sub‐Saharan Africa
Source: HLA. 2017 Dec 25;91(1):36–51. doi: 10.1111/tan.13180 (PMC5767763; doi:10.1111/tan.13180)
Supplement: Supplementary file 7 — Supplementary Information S07 Potential donors for the DRB1*13:04 allelic conversion [file TAN-91-36-s003.pdf]

Supplementary Information S07

Potential donors for the DRB1*13:04 allelic conversion

List of all potential donors for the allelic conversion proposed to generate DRB1*13:04. *Upstream* and *Downstream* correspond to the number of identical nucleotides between the donor and DRB1*11:02:01, the recipient, upstream and downstream the 6pb long fragment.

**Allele**	**Number of conserved nucleotides**		**Fragment size**
	**Upstream**	**Downstream**	
DRB1*13:154	158	39	197
DRB1*13:66:01	158	39	197
DRB1*13:66:02	158	39	197
DRB1*13:30	155	39	194
DRB1*13:169	158	25	183
DRB1*13:21:01	158	25	183
DRB1*13:21:02	158	25	183
DRB1*13:75	158	25	183
DRB1*13:93	61	114	175
DRB1*08:37	126	39	165
DRB1*08:17	126	25	151
DRB1*13:139	61	87	148
DRB1*13:32	30	114	144
DRB1*13:48	30	114	144
DRB1*04:106	30	87	117
DRB1*13:38	30	87	117
DRB1*13:65	30	87	117
DRB1*04:147	73	25	98
DRB1*04:28	73	25	98
DRB1*12:46	58	39	97
DRB1*15:12	60	33	93
DRB1*03:12	61	25	86
DRB1*14:65	60	25	85
DRB1*04:12	30	39	69
DRB1*04:136	30	39	69
DRB1*04:86	30	39	69
DRB1*08:03:02	30	39	69
DRB1*08:03:03	30	39	69
DRB1*08:03:04	30	39	69
DRB1*08:03:05	30	39	69
DRB1*08:03:06	30	39	69
DRB1*08:10	30	39	69
DRB1*08:12	30	39	69
DRB1*08:14	30	39	69
DRB1*08:18	30	39	69

DRB1*08:23	30	39	69
DRB1*08:27	30	39	69
DRB1*08:29	30	39	69
DRB1*08:32	30	39	69
DRB1*08:35	30	39	69
DRB1*08:36:01	30	39	69
DRB1*08:36:02	30	39	69
DRB1*08:38	30	39	69
DRB1*08:40	30	39	69
DRB1*08:46	30	39	69
DRB1*08:53	30	39	69
DRB1*08:56	30	39	69
DRB1*08:58	30	39	69
DRB1*08:60N	30	39	69
DRB1*08:62	30	39	69
DRB1*08:65	30	39	69
DRB1*08:66	30	39	69
DRB1*08:68	30	39	69
DRB1*08:71	30	39	69
DRB1*08:74	30	39	69
DRB1*13:03:01	30	39	69
DRB1*13:03:02	30	39	69
DRB1*13:03:05	30	39	69
DRB1*13:03:06	30	39	69
DRB1*13:101	30	39	69
DRB1*13:115	30	39	69
DRB1*13:12:01	30	39	69
DRB1*13:12:02	30	39	69
DRB1*13:12:03	30	39	69
DRB1*13:12:04	30	39	69
DRB1*13:120	30	39	69
DRB1*13:13	30	39	69
DRB1*13:151	30	39	69
DRB1*13:152	30	39	69
DRB1*13:167	30	39	69
DRB1*13:174	30	39	69
DRB1*13:188	30	39	69
DRB1*13:194	30	39	69
DRB1*13:216	30	39	69
DRB1*13:219	30	39	69
DRB1*13:33:01	30	39	69
DRB1*13:33:02	30	39	69
DRB1*13:33:03	30	39	69
DRB1*13:58	30	39	69
DRB1*13:81	30	39	69
DRB1*13:89	30	39	69

DRB1*13:94:01	30	39	69
DRB1*13:94:02	30	39	69
DRB1*13:95	30	39	69
DRB1*14:63	30	39	69
DRB1*14:78	30	39	69
DRB1*13:133	30	38	68
DRB1*08:33	28	39	67
DRB1*13:161	27	39	66
DRB1*04:107	30	33	63
DRB1*04:125	30	33	63
DRB1*13:03:03	23	39	62
DRB1*13:118	18	39	57
DRB1*01:66	30	25	55
DRB1*04:05:01	30	25	55
DRB1*04:05:02	30	25	55
DRB1*04:05:03	30	25	55
DRB1*04:05:04	30	25	55
DRB1*04:05:07	30	25	55
DRB1*04:05:08	30	25	55
DRB1*04:05:09	30	25	55
DRB1*04:05:10	30	25	55
DRB1*04:05:11	30	25	55
DRB1*04:05:13	30	25	55
DRB1*04:05:14	30	25	55
DRB1*04:05:15	30	25	55
DRB1*04:05:16	30	25	55
DRB1*04:05:17	30	25	55
DRB1*04:09	30	25	55
DRB1*04:10:01	30	25	55
DRB1*04:10:02	30	25	55
DRB1*04:10:03	30	25	55
DRB1*04:103	30	25	55
DRB1*04:104	30	25	55
DRB1*04:11:01	30	25	55
DRB1*04:116	30	25	55
DRB1*04:126	30	25	55
DRB1*04:131:01	30	25	55
DRB1*04:131:02	30	25	55
DRB1*04:137	30	25	55
DRB1*04:152	30	25	55
DRB1*04:17:01	30	25	55
DRB1*04:17:02	30	25	55
DRB1*04:173	30	25	55
DRB1*04:178N	30	25	55
DRB1*04:191	30	25	55
DRB1*04:201	30	25	55

DRB1*04:202	30	25	55
DRB1*04:203	30	25	55
DRB1*04:207	30	25	55
DRB1*04:213	30	25	55
DRB1*04:214N	30	25	55
DRB1*04:24	30	25	55
DRB1*04:45	30	25	55
DRB1*04:48	30	25	55
DRB1*04:67	30	25	55
DRB1*04:77	30	25	55
DRB1*04:80	30	25	55
DRB1*04:81N	30	25	55
DRB1*04:83	30	25	55
DRB1*04:84	30	25	55
DRB1*04:87	30	25	55
DRB1*04:89	30	25	55
DRB1*04:90	30	25	55
DRB1*04:91	30	25	55
DRB1*08:01:01	30	25	55
DRB1*08:01:02	30	25	55
DRB1*08:01:04	30	25	55
DRB1*08:01:05	30	25	55
DRB1*08:01:06	30	25	55
DRB1*08:05	30	25	55
DRB1*08:06	30	25	55
DRB1*08:16	30	25	55
DRB1*08:22	30	25	55
DRB1*08:26	30	25	55
DRB1*08:39	30	25	55
DRB1*08:43	30	25	55
DRB1*08:48	30	25	55
DRB1*08:50	30	25	55
DRB1*08:54	30	25	55
DRB1*08:55	30	25	55
DRB1*08:64	30	25	55
DRB1*08:70	30	25	55
DRB1*08:77	30	25	55
DRB1*08:79	30	25	55
DRB1*10:05	30	25	55
DRB1*13:108	30	25	55
DRB1*13:134	30	25	55
DRB1*13:164	30	25	55
DRB1*13:49	30	25	55
DRB1*13:55	30	25	55
DRB1*13:88	30	25	55
DRB1*14:13	30	25	55

DRB1*14:169	30	25	55
DRB1*14:170	30	25	55
DRB1*14:85	30	25	55
DRB1*04:05:05	28	25	53
DRB1*04:162	27	25	52
DRB1*04:11:02	26	25	51
DRB1*04:11:04	26	25	51
DRB1*04:30	24	25	49
DRB1*08:76	30	19	49
DRB1*13:03:04	8	39	47
DRB1*08:78N	30	16	46
DRB1*04:29	18	25	43
DRB1*13:90	2	39	41
DRB1*08:49	30	9	39
DRB1*08:61	30	7	37
DRB1*08:47	30	5	35
DRB1*09:08	30	5	35
DRB1*04:57	30	4	34
DRB1*08:51	30	2	32
DRB1*13:122	30	2	32
DRB1*08:69	30	1	31
DRB1*04:05:06	1	25	26
